# Augmenter of Liver Regeneration (*alr*) Promotes Liver Outgrowth during Zebrafish Hepatogenesis

**DOI:** 10.1371/journal.pone.0030835

**Published:** 2012-01-26

**Authors:** Yan Li, Muhammad Farooq, Donglai Sheng, Chanchal Chandramouli, Tian Lan, Nilesh K. Mahajan, R. Manjunatha Kini, Yunhan Hong, Thomas Lisowsky, Ruowen Ge

**Affiliations:** 1 Department of Biological Sciences, National University of Singapore, Singapore, Singapore; 2 Department of Zoology, College of Science, King Saud University, Riyadh, Kingdom of Saudi Arabia; 3 Department of Biochemistry and Molecular Biology, Medical College of Virginia, Virginia Commonwealth University, Richmond, Virginia, United States of America; 4 multiBIND biotec GmbH, Köln, Germany; Laboratoire Arago, France

## Abstract

Augmenter of Liver Regeneration (ALR) is a sulfhydryl oxidase carrying out fundamental functions facilitating protein disulfide bond formation. In mammals, it also functions as a hepatotrophic growth factor that specifically stimulates hepatocyte proliferation and promotes liver regeneration after liver damage or partial hepatectomy. Whether ALR also plays a role during vertebrate hepatogenesis is unknown. In this work, we investigated the function of *alr* in liver organogenesis in zebrafish model. We showed that *alr* is expressed in liver throughout hepatogenesis. Knockdown of *alr* through morpholino antisense oligonucleotide (MO) leads to suppression of liver outgrowth while overexpression of *alr* promotes liver growth. The small-liver phenotype in *alr* morphants results from a reduction of hepatocyte proliferation without affecting apoptosis. When expressed in cultured cells, zebrafish Alr exists as dimer and is localized in mitochondria as well as cytosol but not in nucleus or secreted outside of the cell. Similar to mammalian ALR, zebrafish Alr is a flavin-linked sulfhydryl oxidase and mutation of the conserved cysteine in the CxxC motif abolishes its enzymatic activity. Interestingly, overexpression of either wild type Alr or enzyme-inactive Alr^C131S^ mutant promoted liver growth and rescued the liver growth defect of *alr* morphants. Nevertheless, *alr* ^C131S^ is less efficacious in both functions. Meantime, high doses of *alr* MOs lead to widespread developmental defects and early embryonic death in an *alr* sequence-dependent manner. These results suggest that *alr* promotes zebrafish liver outgrowth using mechanisms that are dependent as well as independent of its sulfhydryl oxidase activity. This is the first demonstration of a developmental role of *alr* in vertebrate. It exemplifies that a low-level sulfhydryl oxidase activity of Alr is essential for embryonic development and cellular survival. The dose-dependent and partial suppression of *alr* expression through MO-mediated knockdown allows the identification of its late developmental role in vertebrate liver organogenesis.

## Introduction

Augmenter of Liver Regeneration (ALR), also known as Hepatopoietin (HPO) and growth factor ERV1-like (GFER), is a protein highly up-regulated during liver regeneration and stimulates hepatocyte proliferation. ALR was first purified and cloned from rat liver as a secreted protein of 125 amino acids [1]. The human ortholog of the yeast Essential for Respiration and Viability 1 (Erv1) was identified in 1995 [2] and subsequently purified and cloned from human fetal liver [2], [3] and was also named hepatopoietin (HPO). Erv1 a sulfhydryl oxidase localized in the intermembrane space in mitochondria and is essential for yeast cell survival. In yeast, Erv1 is also involved in Fe/S cluster formation in proteins and Fe homeostasis [4]. Mammalian ALR contains a conserved sulfhydryl oxidase enzymatic domain (ERV1 domain) at the C-terminus and functions as a sulfhydryl oxidase facilitating disulfide bond formation in proteins [5]. To date, homologous ALR proteins have been found throughout the eukaryotic kingdom from fungi to man, suggesting its role in common and important functions. While the enzymatic domain at the C-terminus is conserved, the N-terminal region is highly variable among ALRs in different species, implicating potentially distinct functions of this protein in different species. In both yeast and human, the mitochondria protein Mia40 and cytochrome c have been identified as direct *in vivo* substrates of Erv1/ALR [6], [7], [8]. Whether ALR has additional *in vivo* substrates inside mitochondria or at other subcellular locations is still a mystery.

In mammals, ALR has an additional function, i.e. stimulating hepatocyte proliferation and liver regeneration as a cytokine. In adult rat liver, ALR is believed to be predominantly and constitutively produced and stored in hepatocytes in an inactive form. Upon partial hepatectomy or other hepatic damage, ALR is activated and secreted out of hepatocytes into circulation [9]. As a cytokine, ALR stimulates Mitogen-Activated Protein Kinase (MAPK) pathway by binding to the ALR receptor specifically expressed on hepatocyte cell surface [10]. However, the identity of the cell surface ALR receptor is not yet known. Intracellularly, ALR binds to Jun Activation domain-Binding protein 1 (JAB 1) and potentiates Activator Protein-1 (AP-1) transcription activation pathway utilizing its sulfhydryl oxidase activity [11], [12]. ALR is therefore been called a “cytozyme”, possessing both cytokine and enzyme functions. Nevertheless, it is not clear if the cytokine activity of ALR is dependent on its enzymatic activity.

Recently, the first human disease due to *ALR* R194H mutation has been identified as an autosomal-recessive infantile mitochondrial disorder presenting myopathy with cataract and combined respiratory-chain deficiency [13]. The crystal structure of short form human ALR (sfALR) indicated that R194 is located at the subunit interface, close to the intersubunit disulfide bridges [14]. In vitro characterization indicated that R194H mutation affected the stability of both the long form and short form of human ALR, leading to a significant increase in conformational flexibility [14].

Despite many studies demonstrating various functions of ALR during liver regeneration, its developmental role has not been studied. Based on the fact that ALR exists in large amount in fetal livers of both rat and human [3], [15], [16], we hypothesized that this protein may have crucial roles in liver organogenesis during vertebrate embryonic development.

Zebrafish offers great promise as a model organism to study embryonic liver development and liver diseases [17]. Significantly, zebrafish adult liver regenerates efficiently similar to mammals [18]. However, different from mammals, zebrafish embryonic liver is not a hematopoiesis organ, thus liver organogenesis could be studied independently from the defects caused by hematopoietic deficiencies [19].

In vertebrate, liver develops from the anterior endoderm. In both zebrafish and mammals, Fgfs, Bmps, and the Wnt/β-catenin pathway are the primary signaling pathways required for zebrafish hepatogenesis (reviewed in [18]). In zebrafish, cells in the anterior endodermal rod become specified to the hepatic cell fate at around 22 hours post fertilization (hpf). Both Fgf and Bmp signaling are essential for hepatic specification [20]. On the other hand, Wnt2bb produced from the adjoining mesoderm cells is involved in multiple stages of hepatogenesis including hepatic specification, hepatocyte differentiation and liver outgrowth [21]. By 26–28 hpf, hepatoblasts migrate and thicken on the left side of the anterior gut tube to form the liver bud, marking the beginning of the budding phase of liver formation. Transcription factors such as *hnf4*, *hhex*, and *prox1* are expressed in the liver bud at this stage. Beyond 32 hpf, liver bud begins to express differentiated hepatocyte markers such as ceruloplasmin (*cp*), transferrin (*tfa*), and liver fatty acid binding protein (*lfabp*). Around 50 hpf liver bud completely delaminates from gut tube. Subsequently, liver enters a rapid growth phase during which it becomes vascularized and expands rapidly. By 5 days post fertilization (dpf), liver has crossed the midline to reach the right side of the body [19].

Despite the significant advances in our understanding of vertebrate hepatogenesis in recent years, our knowledge of the molecular mechanisms and genes involved are far from complete. In this work, we showed for the first time that the zebrafish Alr is a hepatocyte mitogen during liver organogenesis. Knockdown of *alr* interfered with liver expansion, resulting in a small liver phenotype. Intriguingly, zebrafish Alr controls liver development possibly through sulfhydryl oxidase dependent as well as independent signaling pathways. This is the first study which categorically identifies a crucial role for Alr in vertebrate hepatogenesis.

## Materials and Methods

### Ethics statement and zebrafish lines

Fish maintenance and experimental protocols were approved by Institutional Animal Care and Use Committee (IACUC) of National University of Singapore (Protocol 007/06). Embryos were collected and staged as described [22], [23]. The transgenic line used is *Tg(lfabp:DsRed; elaA:EGFP)* [24] in which DsRed is expressed in liver and EGFP is expressed in exocrine pancreas.

### Molecular cloning of zebrafish *augmenter of liver regeneration* (*alr*)

The full length cDNA of zebrafish *alr* (NM_001089386.1) was obtained by high fidelity RT-PCR using Advantage High Fidelity 2 (HF2) PCR kit (Clontech) using pools of RNA from mixed stages of zebrafish embryos. The PCR product was cloned into pGEM-T vector (Promega) and validated by sequencing. The *alr* gene was subcloned into pCS2(+) (Addgene) vector for *in vitro* transcription. The *alr* ORF was cloned into pEF6/V5-His-TOPO (Invitrogen) vector to transiently express Alr with a C terminal V5 epitope in cultured cells. After PCR addition of the restriction enzyme sites on zebrafish *alr* coding sequence, the cDNA was cloned back into pGEM-T vector (Promega), and subcloned into pEGFP-N1 (Clontech) vector to create an ALR-EGFP fusion protein. Cys 131 of zebrafish Alr was mutated into Ser by QuickChange site-directed mutagenesis kit (Stratagene) in pCS2-*alr* plasmid. Both the wild type and mutant *alr* genes are cloned into pET28b vector (Novagen), with a N-terminal His-tag for protein expression and purification from *E.coli*.

### Whole mount in situ hybridization (WISH)

WISH was performed using digoxigenin labeled RNA antisense probe for the following genes: *cp*, *prox1*, *alr*, *insulin*, *foxa3* according to the Zebrafish Book [23]. The embryos were grown in 0.003% 1-phenyl-2-thiourea (PTU) solution to block pigmentation.

### Knockdown of *alr* by antisense morpholino injection

Morpholino antisense oligonucleotides were purchased from Gene Tools, and were dissolved in sterile water at the concentration of 1 mM. A total amount of 5–10 ng morpholino per embryos was injected into either *Tg (lfabp:DsRed; elaA:EGFP)* or local wild type embryos to monitor liver formation. The *alr* morpholinos used are:

1. Translation blocking morpholino (ATG morpholino): 5′-CGTGTGCAGCTGCCATGTTGTTATG, 5 bp mismatch control: 5′-CcTGTGgAGCTcCCATcTTcTTATG;
2. Splicing inhibiting morpholino targeting first exon and first intron splicing junction (E1I1 morpholino): 5′-TCATTCATAATTGTTCACCTGCACC, 5 bp mismatch control: 5′-TgATTgATAATTcTTCAgCTcCACC;
3. Splicing inhibiting morpholino targeting first intron and second exon splicing junction (I1E2 morpholino): 5′-CTCTCCTGTACAACATATCACGTTG, 5 bp mismatch control: 5′-CTCTCgTcTACAAgATATgACcTTG.

### Total RNA extraction and RT-PCR

Total RNA from zebrafish embryos and adult tissues was extracted using TRI reagent (Ambion) following manufacturer's instruction. Adult zebrafish tissues samples were extracted from a pool of 5–10 fishes. The livers were extracted from 100 five-days old fishes, 50 two-week old fishes, 30 three-week old fishes, 20 four-week old fishes, 10 six-week old fishes and 5 adult fishes (three-month and nine-month old fishes) respectively. For fishes younger than 5 dpf, total RNA were extracted from a pool of 30–50 embryos.

RT-PCR was performed using one-step RT-PCR kit (Qiagen) and 0.5 µg total RNA per reaction. The primers used to amplify *alr* were: foward 5′-GGGTCGTCTCCACATAGC-3′ and reverse 5′-CTCTCCATCGCTCATCCACCCT-3′. The one-step RT-PCR conditions were: 50°C 30 min; 95°C 15 min; 95°C 30 sec, 55°C 30 sec, 72°C 40 sec, for 30 cycles; 72°C 5 min. The zebrafish ribosomal protein S18 (*rps18*) or *β-actin* were used as internal control. The cycle numbers used for each gene are selected within the exponential amplification phase of that gene. The relative signal intensity of *alr* bands were determined using the ImageJ software by normalizing to the respective *rps18* band.

### Generation of 5′ capped mRNA by in vitro transcription

5′-capped mRNA of zebrafish *alr* and *alr* ^C131S^ were synthesized using mMessenger mMachine kit (Ambion) and injected into fertilized embryos at 1–2 cell stages. For overexpression, 1.6 ng *alr* mRNA was injected into each 1–2 cell stage embryos. To rescue morphants, 5 ng E1I1 morpholino and 1.6 ng *alr* mRNA were co-injected into 1–2 cell stage zebrafish embryos.

### Liver size quantification for *alr* overexpression and morphant rescue

Embryos at 48 hpf that have gone through *prox1* WISH to label the liver were used for liver size quantification. Photos were taken for these embryos from dorsal view by microscope under the same magnification and then analyzed in Photoshop CS3 software. The liver size in 2-D dimension was represented by the number of pixels in liver region. Data was presented as mean ± standard deviation (SD). Student's t-test was used to analyze the data and p<0.05 is considered significant.

### Immunostaining for proliferation and TUNEL assay

Embryos were fixed in 4% paraformaldehyde at 4°C for overnight. Frozen sections of 10 µm were collected. After blocking with 3% BSA for 1 h at room temperature, the sections were incubated with rabbit anti-proliferating cell nuclear antigen (PCNA) (1∶250 dilution, Santa Cruz) or rabbit anti-phospho histone H3 (p-H3) antibody (1∶100 dilution, Millipore) at 4°C overnight. Secondary antibody of Alexa Fluor 568 conjugated anti-rabbit IgG (Invitrogen) was then incubated for 1 h at room temperature. The stainings were imaged with fluorescent microscope.

To calculate the percentage of PCNA/p-H3 positive hepatocytes per embryo, number of stained hepatocytes and total hepatocytes were counted on each section. For PCNA staining of 4 dpf embryos, 3 livers, 7 sections per liver were counted. For p-H3 staining of 4 dpf embryos, 4 livers, 7 sections per liver were counted. For p-H3 staining of 48 hpf embryos, 5 livers, 7 sections per liver were counted. Data was presented as bar graph of mean ± standard deviation (SD) and p<0.05 was considered significant as analyzed by student's t-test.

TUNEL assay was performed using Roche in situ cell death detection kit following the manufacture's instruction and quantified similarly.

### Cellular localization study of zebrafish Alr

The expression plasmids for Alr-V5 and Alr-EGFP were transfected into human hepatocellular carcinoma cells (HepG2) and zebrafish liver cells (ZFL) [25] using FuGENE HD transfection reagent (Roche), into human embryonic kidney cells (HEK293T) cells using TransIT-LT1 transfection reagent (Mirus). The transfected cells were labeled with MitoTracker Red (Invitrogen) before being fixed in 4% PFA, followed by immunofluorescent staining using mouse anti-V5 primary antibody (1∶500 dilution, Invitrogen) and Alexa Fluo 488 anti-mouse IgG (1∶1000 dilution, Invitrogen).

For western blot analysis, cells were lysed using RIPA lysis buffer at 48 hour post transfection. The culture medium was collected and cold acetone was used to precipitate proteins from the medium. Mitochondria isolation from the cultured cells was carried out using Mitochondria Isolation Kit for Cultured Cells (Pierce).

### Western blot

Western blot was performed using standard method and probed with mouse anti-V5 antibody (Invitrogen), mouse anti-GFP antibody (Millipore), rabbit anti-VDAC/porin antibody (Santa Cruz), mouse anti-α tubulin (Sigma) and mouse anti-β actin (Santa Cruz) respectively.

### Recombinant Alr expression and purification from *E.coli*

The wild type pET28b-alr and mutant pET28b-alr^C131S^ were expressed in *E. coli* BL21-DE3 strain. Bacterial pellet were collected, lysed in lysis buffer, and soluble proteins were subjected to Ni-NTA resin purification under native condition (Promega). Imidazole in the purified protein solution was removed by dialysis.

### Sulfhydryl oxidase enzymatic assay

Lysozyme (Sigma) was reduced and used as substrate as described before [5]. Reduced glutathione and DTT were also used as substrates. The ability of Alr to introduce disulfide bonds into the substrates were measured by Ellman's reagent (Sigma) which can quantify the number of free thiol groups as described by Lisowsky et al. [5]. The enzymatic reactions were carried out at room temperature.

## Results

### *alr* expression in zebrafish adult tissues and during embryogenesis

The zebrafish *alr* cDNA was cloned by 5'RACE from local wild type embryos, and it codes for a protein of 191 amino acids. Sequence alignment showed that zebrafish Alr protein was 62% identical to human and mouse short form ALR, 48% identical with their long forms (Fig. S1). Phylogenetic and synteny analyses showed that this zebrafish *alr* was the ortholog of the mammalian *ALR* as well as the yeast ERV1 (Fig. S2).

**Figure 1:**
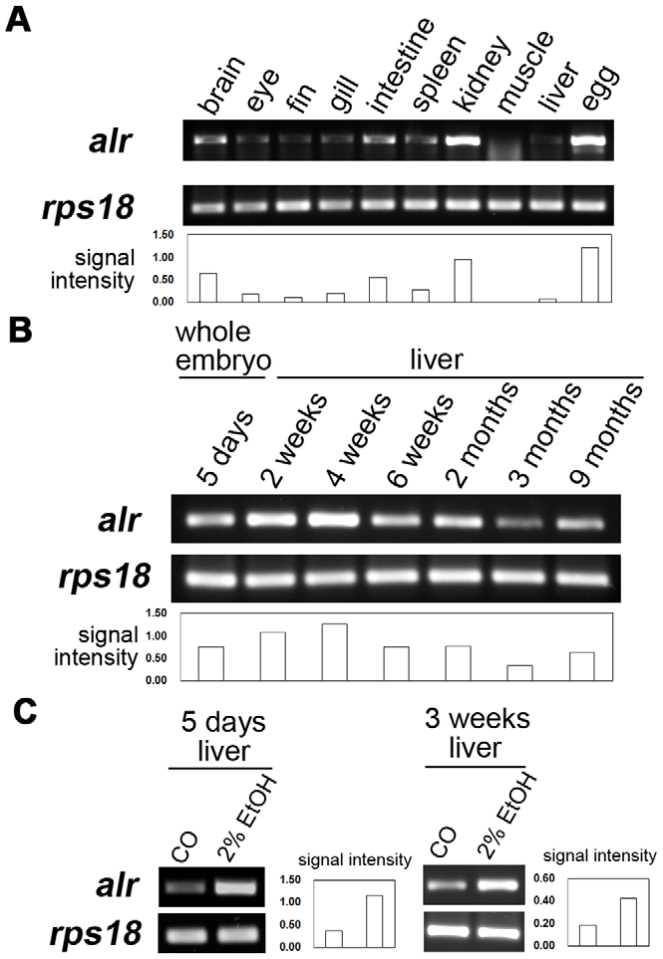
Expression of zebrafish *alr* in adult tissues, livers of fishes of various ages and its response to alcohol treatment. A. *alr* mRNA expression in zebrafish adult tissues analyzed by semi-quantitative RT-PCR. Kidney and egg have the highest *alr* expression level (upper panel). Low expression was detected in liver. The ribosomal protein S18 (*rps18*) (lower panel) was used as the internal loading standard. B. Liver *alr* mRNA expression in fishes of various ages. *alr* is expressed at high levels in 2–4 weeks old fishes, but reduces significantly in 6 weeks old fishes. The expression level is further reduced in adult fishes (3–9 months old). *alr* expression in 5 dpf whole embryo is used as a comparison. C. Ethanol treatment significantly increased *alr* expression in livers of 5 dpf larvae and 3 weeks old young fish. Fish was treated with 2% ethanol for 32 hours, a condition previously shown to potently induce hepatic steatosis in zebrafish embryonic liver [33]. The relative signal intensity of all the *alr* bands were shown beside the corresponding gel photos.

**Figure 2:**
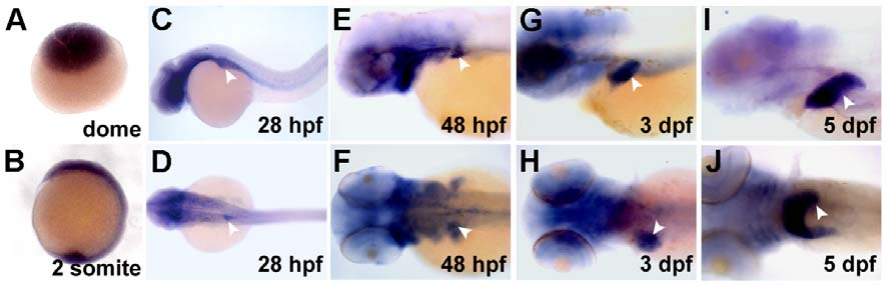
Expression of *alr* during zebrafish embryonic development. Whole mount in situ hybridization (WISH) shows expression of *alr* mRNA at different embryonic stages. During early stages, expression of *alr* is ubiquitous (A, B). Expression in the brain and pharyngeal arches are also observed (C, D, J). From 28 hpf onwards, the expression of *alr* is detected in liver (white arrow head) throughout hepatogenesis (C–J). C, E, G, I: lateral view, anterior to the left; D, F, H, J: dorsal view, anterior to the left.

To understand the function of zebrafish *alr*, we first determined its spatial and temporal expression pattern in adult zebrafish tissues as well as in embryos of various developmental stages. Semi-quantitative RT-PCR revealed that *alr* was expressed at different levels in various adult tissues, with the highest expression in kidney and egg (Fig. 1A). The high abundance of *alr* mRNA in eggs indicates that *alr* is present as maternal mRNA and may play important roles in early embryonic development. Intermediate level expression of *alr* can be detected in brain and intestine. A low level *alr* expression can be detected in adult liver, spleen, gill, eye and fin while muscle showed almost no detectable *alr* mRNA. Liver *alr* expression is highest in embryos and young fish and gradually declined to a moderate level as the fish get older (Fig. 1B and Fig. 2). In comparison, relatively high level of Alr expressions have been reported in livers of adult rat and human [1], [9], [26].

It is known that upon partial hepatectomy in rat, serum ALR levels increase with concomitant decrease in hepatic ALR protein, suggesting that ALR is released by the liver after PH [9]. Expression of ALR is also increased in acute or chronic human liver diseases such as fibrosis and cirrhosis, as well as in liver carcinoma [26], [27], [28], suggesting liver protective functions of ALR in liver diseases. Indeed, ALR has been shown to function as a survival factor for hepatocytes and depletion of ALR protein by antisense oligonucleotide leads to hepatocyte cell death [29]. Acute liver damage induced by toxins, such as ethanol, is known to stimulate hepatic stimulatory substance (HSS) activity in the injured livers, and exogenous HSS administration increased the injured liver hepatic proliferation post toxin treatment [30], [31]. ALR is a purified protein of HSS [1] and has been reported to stimulate hepatocyte proliferation directly as well as indirectly through Kupffer cells [3], [32]. We therefore investigated if Alr is up-regulated by alcohol induced acute liver injury. Indeed, when zebrafish embryo (5 dpf) and young fish (3-weeks old) were treated with 2% ethanol, a condition previously shown to induce hepatic steatosis (fatty liver) in zebrafish [33], *alr* expression was significantly up-regulated in the liver (Fig. 1C). This result indicates that liver injury can induce Alr expression in the liver of zebrafish larvae, similar to the behavior of mammalian ALR after liver injury. However, the role of Alr in zebrafish liver steatosis is unclear at this stage.

Temporal and spatial expression of *alr* analyzed by WISH indicated a ubiquitous presence of *alr* mRNA in early stage embryos (Fig. 2A). During the segmentation period, it is highly expressed in the ventral portion of the brain (Fig. 2, B–D). Notably, *alr* mRNA is expressed in the developing liver from the liver budding stage (28 hpf) and the liver expression persists and become intensified during the liver growth phase from 3–5 dpf (days post fertilization) (Fig. 2, C–J, white arrow). In addition, *alr* is also expressed in brain, pharyngeal arches and exocrine pancreas during the liver growth phase (Fig. 2G–J and Fig. S3). The high *alr* expression in the developing liver throughout hepatogenesis suggests that *alr* might play an important role in liver organogenesis.

### *alr* promotes liver outgrowth during zebrafish hepatogenesis

**Figure 3:**
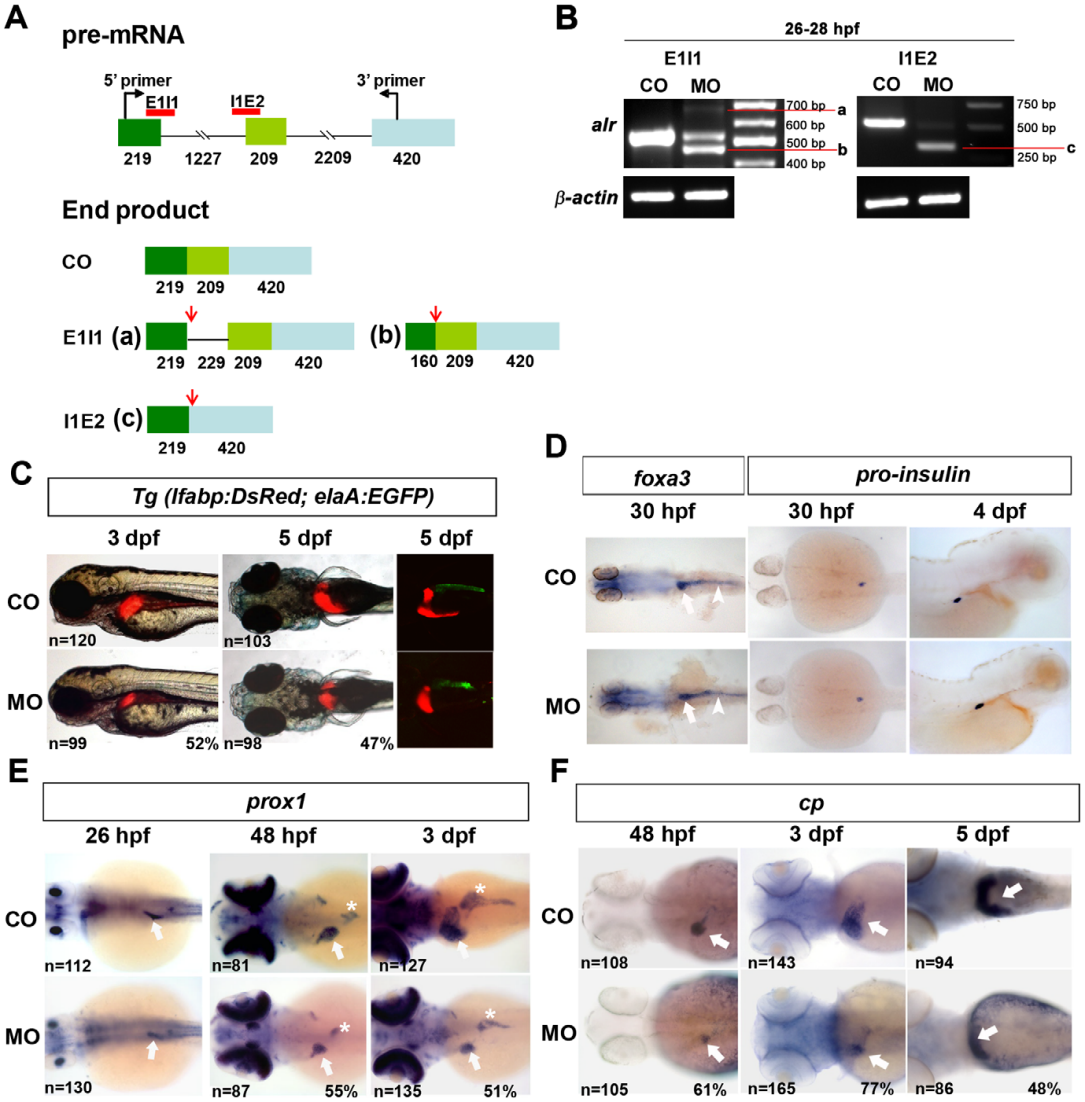
Knockdown of *alr* by antisense morpholino oligonucleotide inhibits liver growth. A. Schematic presentation of *alr* pre-mRNA and morpholino design. *alr* pre-mRNA consists of 3 exons (shown by squares) and 2 introns (shown by lines). The number of nucleotides in each region is labeled below the region. The red lines indicate the targeting sites of the two splicing inhibiting morpholinos, E1I1 and I1E2. In 5-bp mismatch control morpholino injected embryos (CO), splicing of *alr* pre-mRNA is not affected. In E1I1 morpholino injected embryos, two alternative splicing sites are used. One of the alternative splicing sites is at 229 bp downstream of the 5′ boarder of intron 1, generating mRNA product a; the other alternative splicing site is in exon 1, 160 bp downstream of the 5′ end of exon 1, producing the mRNA product b. In I1E2 morphants, E1I1 splicing site and I2E3 splicing site will join together and generate mRNA c (with exon 2 removed). The red arrows show the stop codons present in these alternatively spliced mRNAs. B. RT-PCR results demonstrate the potent knockdown of endogenous *alr* mRNA by the splicing morpholinos. Bands a, b and c are described in Fig. 3A. Morpholinos were injected at 5 ng per embryo, and total RNA was extracted from these embryos at 26–28 hpf. CO, 5-bp mismatch morpholino injected embryos; MO, morpholino injected embryos. β-actin was used as internal control for RT-PCR. C. Knockdown of *alr* suppressed liver growth in *Tg(lfabp:DsRed;elaA:EGFP)* embryos. Three morpholinos showed similar phenotype, and the photos shown are from translation blocking morpholino injected embryos. Liver size (red color) was reduced significantly in MO, compared to CO. In the right panel, confocal fluorescent images show suppressed liver (red) and exocrine pancreas (green). All images are anterior to the left, side view for 3 dpf embryos, dorsal view for 5 dpf embryos. D. Knockdown of *alr* did not affect intestine and endocrine pancreas formation. Intestine was marked by WISH using pan-endoderm marker *foxa3*. Endocrine pancreas was shown by WISH using *pro-insulin* marker. Dorsal view, anterior to the left for 30 hpf embryos. Side view, anterior to the right for 4 dpf embryos. White arrow points to liver bud, White arrow head points to intestine. E. Liver formation in *alr* morphants monitored by hepatoblast marker *prox1*. In *alr* morphants, an obviously reduced liver size was observed at 48 hpf and 3 dpf. Although a discernible small liver was also observed in some embryos at 26 hpf, but quantification of embryo population failed to show a statistically significant difference comparing to controls. The number of embryos analyzed was shown on the bottom left of each panel while the percentage of embryos with small liver was labeled on the bottom right corner. White arrow points to liver, white star points to pancreas. All images are dorsal view, anterior to the left. F. Liver formation in *alr* morphants monitored using hepatocyte marker *cp*. In *alr* morphants, a reduced liver size was also observed at 48 dpf, 3 dpf and 5 dpf. White arrow point to liver. All images are dorsal view, anterior to the left.

To investigate the developmental functions of *alr* in zebrafish, morpholino antisense oligonucleotide (morpholino) mediated gene knockdowns were performed. As illustrated in Fig. 3A, zebrafish *alr* gene has three exons separated by two introns. Three morpholinos were designed, targeting the translation starting site (ATG), exon1-intron1 boundary (E1I1) and intron1-exon2 boundary (I1E2) respectively. Morpholinos were microinjected into 1–2 cell stage embryos, using 5 bp mismatch morpholinos as controls.

Both splicing-blocking morpholinos E1I1 and I1E2 potently knocked down the endogenous *alr* mRNA expression in a dose-dependent manner. At 26–28 hpf, a stage in which liver has just budded from the anterior endoderm and *alr* is expressed in the budding liver, significant reductions of endogenous *alr* mRNA were demonstrated in morphants injected with 5 ng morpholino per embryo (Fig. 3B). Nevertheless, low level endogenous *alr* mRNAs are still present in morphants at this morpholino dose (Fig. 3B). The predicted splicing product in E1I1 morphants (Fig. 3A and 3B, product a) as well as an alternative splicing product using an upstream splicing donor site (Fig. 3A and 3B, product b) were detected (Fig. 3B). The I1E2 morphants generated a predicted aberrant RNA product lacking the second exon which carries premature stop codons (Fig. 3A and 3B, product c).

When injected at ≥10 ng morpholino per embryo, embryos showed severe morphological defects including a curved body, small head with high level of apoptosis (especially in brain), no circulation, and cardiac edema (data not shown). In comparison, embryos injected with the same amount of 5 bp mismatch control morpholino did not produce such morphological defects. This was more obvious with the translation blocking morpholino (data not shown). It therefore seems that the maternally supplied *alr* mRNA plays some fundamental roles in early zebrafish embryonic development. Higher amount of morpholino (10 ng/embryo) leads to death of embryos within 24 hpf. When injected at 5 ng morpholino per embryo, embryos are morphologically normal, except for a mild developmental delay. Thus all functional studies presented in this paper were carried out with this morpholino dose.

The effect of *alr* knockdown on liver formation was monitored using the transgenic line *Tg(lfabp:DsRed; elaA:EGFP)*. In this transgenic line, liver-specific expression of DsRed (red fluorescence) is easily visible after 60 hpf while the exocrine pancreas is labeled green with EGFP from 4 dpf onwards [24]. Knockdown of *alr* lead to an obvious reduction in liver size in morphants from 3–5 dpf compared to control morpholino injected embryos at the same stage (Fig. 3C and Fig. S4). Knockdown of *alr* using three different morpholinos showed similar small liver phenotype (Fig. 3 and data not shown).

Growth of the exocrine pancreas is also inhibited in *alr* morphants (Fig. 3C, right panels; Fig. 3E, middle and right panels, indicated by *), consistent with *alr* expression in this organ (Fig. S3). At 5 dpf, both liver and exocrine pancreas was much smaller in *alr* morphants comparing with the control. The smaller exocrine phenotype is also observed using exocrine pancreas marker *elaB* in WISH (data not shown). In contrast, the endoderm rod marked by *foxa3* and the endocrine pancreas marked by *insulin* were not affected (Fig. 3D).

During zebrafish liver organogenesis, competent endoderm cells become specified into bipotential hepatoblasts upon induction and later differentiate into hepatocytes or cholangiocytes [19]. Subsequent proliferation of hepatocytes and other liver cells underscore the growth of the liver. In order to determine at which stage of liver development *alr* functions, WISH with hepatoblast/hepatocyte markers were performed in *alr* morphants. As shown in Fig. 3, *prox1* (marks hepatoblast/hepatocyte) expression in the liver primordial region was detected at 26 hpf in *alr* morphants, suggesting that specification of liver progenitor cell hepatoblasts was not affected (Fig. 3E). Consistently, the pan-endoderm marker *foxa3* showed the presence of a sickening of the anterior endoderm rod at 30 hpf, indicating liver budding (Fig. 3D). Nevertheless, liver size marked by *prox1* expression is obviously smaller in the morphants at 48 hpf (55% of embryos) and 3 dpf (51% of embryos). In comparison, the development of lens is not affected in *alr* morphants (100% of morphants in all developmental stages examined) despite the high *prox1* expression in lens. Since *prox1* is expressed in hepatoblast and hepatocyte, the small liver phenotype in *alr* morphants suggests the possibilities of *alr* function in differentiation of hepatoblast to hepatocyte or proliferation of hepatocyte.

To distinguish the possibilities, we analyzed the expression of the hepatocyte marker *cp* which is expressed in the liver bud from 32–34 hpf onwards. It is also expressed in the yolk syncytial layer [34]. No significant delay in liver *cp* expression was observed in *alr* morphants (data not shown). From 48 hpf onwards, an obvious reduction of liver size was observed in *alr* morphants using *cp* as a marker while its expression in the yolk syncytial layer is not affected (Fig. 3F).

As a summary of the knockdown experiment, hepatoblast markers (*prox1*, *foxa3*) were present in the liver budding region at 26–30 hpf in *alr* morphants, but marker genes for hepatocytes (*prox1*, *cp* and *lfabp*) showed that the liver is much smaller compared to control between 48 hpf to 5 dpf. Altogether, the above results indicate that *alr* plays a major role in liver outgrowth, but has negligible influence on hepatoblast determination or differentiation to hepatocyte.

### Knockdown of *alr* reduces hepatocyte proliferation without affecting apoptosis

**Figure 4:**
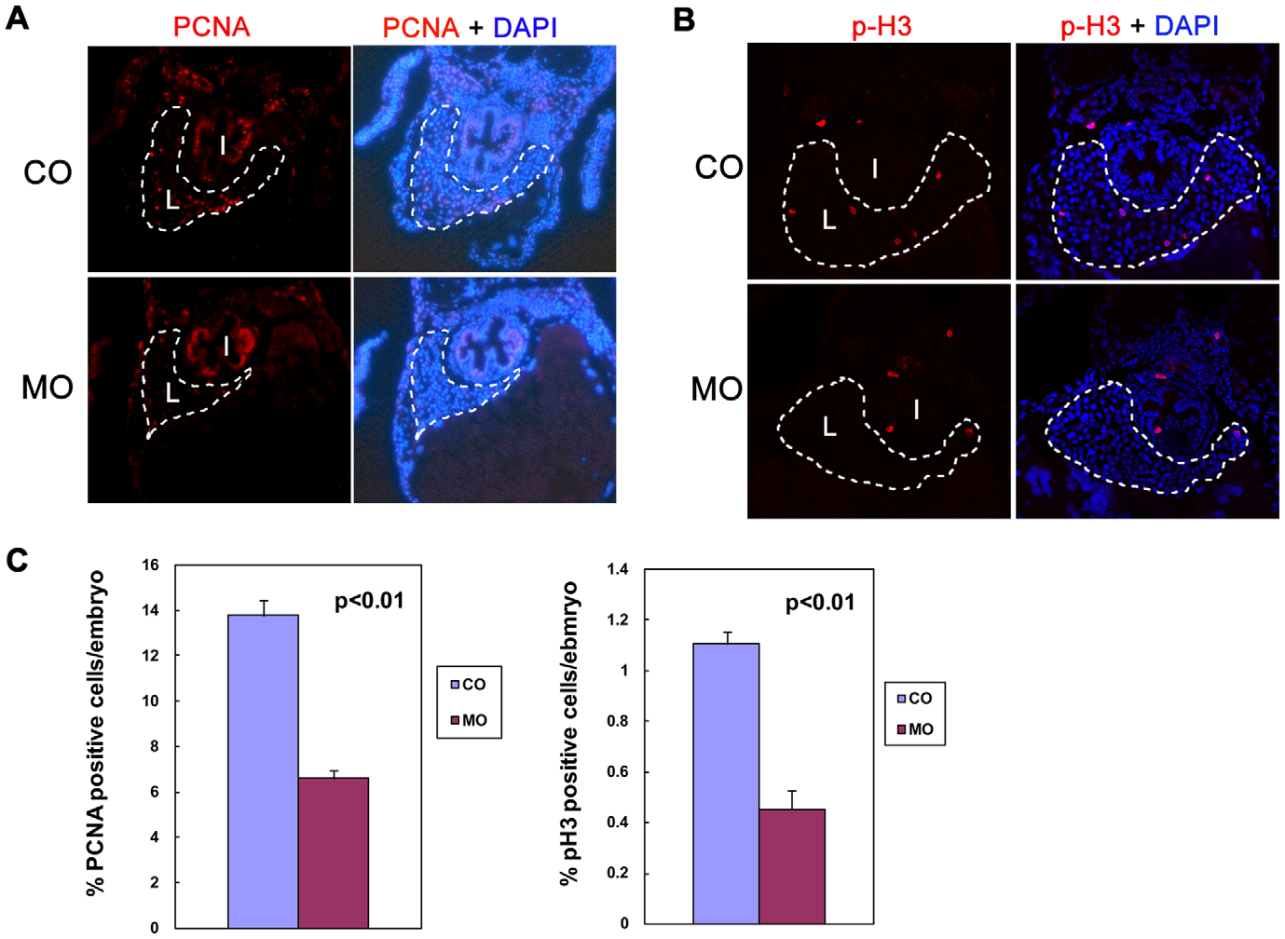
Knockdown of *alr* reduces hepatocyte proliferation. A & B. Hepatocyte proliferation demonstrated by immunofluorescent staining of proliferation markers in 4 dpf embryos: proliferating cell nuclear antigen (PCNA) (A) and phosphor-histone 3 (p-H3) (B). The sections were counterstained with DAPI to label nucleus. PCNA and p-H3 staining is co-localized with DAPI, indicative of nucleus staining. Both PCNA and p-H3 staining showed a significantly reduced hepatocyte proliferation in morphants without affecting proliferation in other tissues such as intestine. I: intestine; L: liver. Dash line circles the boundary of liver. C. Quantification of hepatocyte proliferation. Percentage of PCNA positive hepatocytes in liver is reduced from 13.8% in CO to 6.6% in MO. Percentage of p-H3 positive hepatocytes in liver is reduced from 1.1% in CO to 0.45% in MO. Values are means ± standard deviation (SD). Hepatocytes were counted based on cell morphology.

As hepatocytes are the parenchymal cells in liver which constitute more than 80% of the liver, the small liver phenotype in *alr* morphants could results from reduced hepatocyte proliferation and/or increased apoptosis. To determine the mechanism, we analyzed hepatocyte proliferation by immunofluorescent staining with two commonly used cell proliferation markers: proliferating cell nuclear antigen (PCNA) and phosphorylated histone 3 (p-H3). As shown in Fig. 4, the hepatocyte proliferation rate in the liver of 4 dpf embryo is reduced more than 50% in *alr* morphants compared to control embryos (injected with same amount of 5 bp mismatch control morpholino) using both proliferation markers.

In contrast, no increase in liver cell apoptosis in *alr* morphants was observed as determined by TUNEL assays. A similar low level hepatocyte apoptosis was observed in 4 dpf *alr* morphants and control embryos, with only a couple of cells stained positive on each section (Fig. S5). The low level of apoptosis in the developing liver is consistent with previous report [35]. In comparison, similar TUNEL assay detected high level apoptosis in embryos after heat-shock, a treatment known to induce apoptosis (Fig. S5) [36].

Together, these results demonstrate that *alr* functions as a hepatocyte mitogen and promotes liver growth by stimulating hepatocyte proliferation during zebrafish liver organogenesis.

### Zebrafish Alr is localized in the cytosol and mitochondria

Subcellular localization is important for protein function. In mammals, two protein isoforms of ALR exist: the long form and the short form. While the short from have been shown to be localized in the nucleus, the long form is localized in the intermembrane space of mitochondria and the cytosol [4], [37], [38], [39]. The identity of the secreted Alr isoform is still unclear up to now. *In vitro*, both human ALR125 (short form) and ALR205 (long form) can stimulate hepatoma cell proliferation as an extracellular growth factor [3], [40]. The zebrafish Alr is similar in size to the long form of mammalian ALR as well as the yeast ERV1. No equivalent short form zebrafish Alr has been detected in both embryos and adult zebrafish. Sequence analysis indicated that similar to ALRs in other species, zebrafish Alr does not contain any identifiable signal peptide or typical mitochondria import sequence.

**Figure 5:**
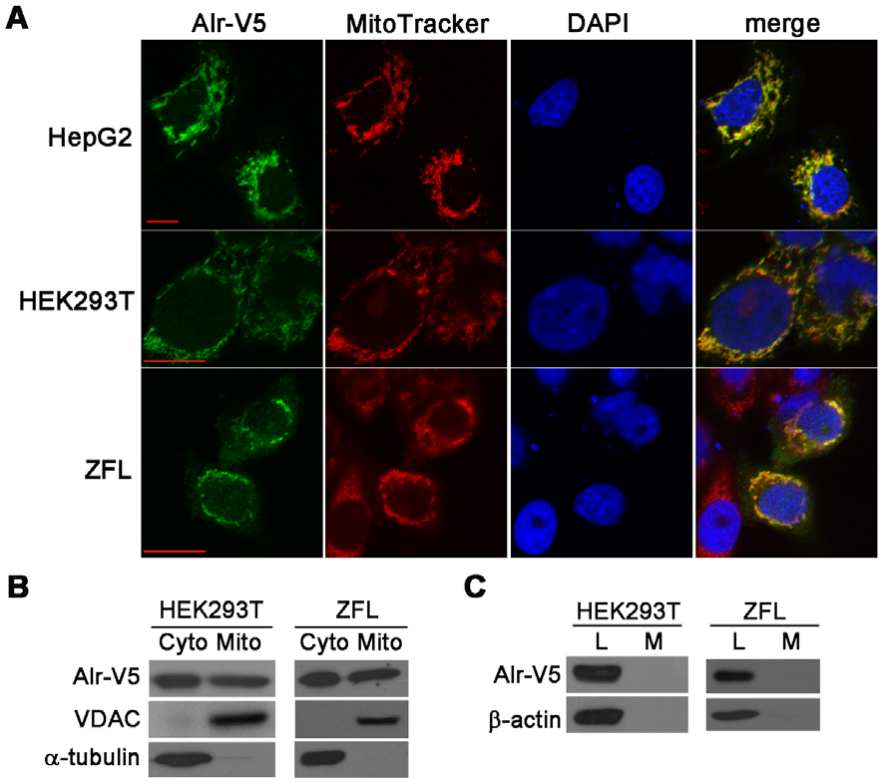
Zebrafish Alr is localized in both the cytosol and mitochondria, but neither in the nucleus nor secreted outside of the cell. A. Alr subcellular localization by immunofluorescent staining. Human hepatocellular carcinoma cells HepG2, human embryonic kidney cells HEK293T and zebrafish liver cells ZFL were transfected with pEF6/V5-His-TOPO plasmid expressing Alr-V5. MitoTracker was used to label the mitochondria and the cells were counter stained with DAPI to mark the nucleus. The Alr protein is co-localized with MitoTracker in the mitochondria, but not present in nucleus. Scale bar is 10 µm. B. Alr subcellular localization by cell fractionation. Western blot revealed that Alr was localized in both the cytosol and mitochondria fractions in transfected HEK293T cells and zebrafish liver cell line (ZFL). Alr was detected by anti-V5 antibody. The mitochondrial porin voltage-dependent anion channel (VDAC) was used as the mitochondria marker while α-tubulin was used as the cytosolic marker. C. Alr was not secreted outside of cell. Alr-V5 was detected in cell lysates but not in the conditioned medium in both HEK293T and ZFL cells. β-actin was used as loading control. L, cell lysate; M, medium.

To determine the subcellular localization of zebrafish Alr, plasmids expressing Alr-V5 and Alr-EGFP fusion proteins were generated and transiently expressed in HepG2 (human hepatocellular carcinoma cell), HEK293T (human embryonic kidney cell) as well as ZFL cells (zebrafish liver cell). Live cell imaging demonstrated that Alr-V5 protein is mainly localized in the cytosol but not in the nucleus (Fig. 5A). Co-localization study using the mitochondria marker MitoTracker demonstrated that Alr-V5 is localized in mitochondria (Fig. 5A). Furthermore, injection of *in vitro* transcribed Alr-EGFP mRNA into 1–2 cell stage zebrafish embryos and detection of Alr-EGFP fusion protein in the 6 hpf embryos by anti-EGFP antibody staining also indicated a predominant cytosol localization of the fusion protein (Fig. S6). Cell fractionation and western blot further revealed that both Alr-V5 and Alr-EGFP are localized in the cytoplasm as well as mitochondria in both HEK293T and ZFL cells. In the same cell fractionation experiment, the mitochondria protein voltage dependent anion channel protein (VDAC) is only present in the mitochondria fraction and α-tubulin is only present in the cytosol, demonstrating the purity of cell fractions isolated (Fig. 5B). No Alr-V5 or Alr-EGFP fusion protein is detected in the medium of transfected HEK293T or ZFL cells (Fig. 5C and Fig. S6). In comparison, high level expression of Alr-V5/Alr-EGFP was observed in the cell lysates. This result indicates that zebrafish Alr is most likely not secreted outside of the cell under normal cell culture conditions. The zebrafish Alr may have functions in both the mitochondria as well as the cytosol.

### Zebrafish Alr is a flavin-linked sulfhydryl oxidase

ALR is known as a “cytozyme”, bearing both cytokine and enzymatic activity. Several members of the ERV1/ALR family are sulfhydryl oxidases including those from human, rat, *Arabidopsis*, and yeast [5], [41], [42]. The importance of sulfhydryl oxidase has been well documented in yeast [43], [44]. The yeast Erv1p, localized in the intermembrane space of mitochondria, forms disulfide relay system with Mia40. Erv1p oxidizes Mia40 to recycle it for oxidative folding of proteins imported to mitochondria. The electron of Erv1p is then transferred to cytochrome C and finally to oxygen. Intracellular human short form ALR (sfALR) binds to Jun Activation domain-Binding protein 1 (JAB-1) and potentiates Activator Protein-1 (AP-1) signalling pathway in a sulfhydryl oxidase dependent manner [12]. On the other hand, extracellular human sfALR activates MAPK pathway and stimulate HepG2 cell proliferation independent of its enzymatic function [12].

**Figure 6:**
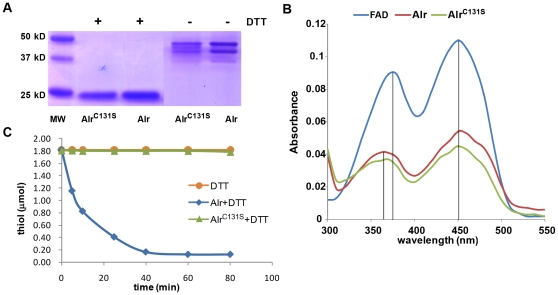
Zebrafish Alr is a flavin-linked sulfhydryl oxidase. A. Alr is present as dimers. Recombinant zebrafish Alr purified from *E.coli* was examined by SDS-PAGE and stained by commassie blue. In the presence of the reducing reagent DTT, Alr protein is in the monomer form, with a size of around 23 kD. In the absence of DTT, both Alr and the mutant Alr^C131S^ are present as dimers with sizes in the 40∼46 kD region. B. Absorption spectra of recombinant zebrafish Alr and Alr^C131S^ protein at 15 µM. Free FAD at 15 µM was used as reference and its spectra show the typical riboflavin spectrum, with two absorbance peaks at around 375 nm and 450 nm. The absorption spectra of both the wild type Alr protein and mutant Alr^C131S^ protein are characteristic of the FAD moiety, with a minor shift of the first peak to 365 nm compared to the free FAD. Under equal molar concentration, the amount of Alr-bound FAD is only half of the free FAD, indicating about 50% loading of FAD in the recombinant Alr preparation. C. Alr is a sulfhydryl oxidase. Enzymatic assay using DTT as substrate, showing the reduction of free thiol groups overtime. The blue line represents DTT alone. Wild type Alr protein oxidized thiol groups over time while the CxxC motif mutant, Alr^C131S^, completely lost the sulfhydryl oxidase activity.

A common characteristic of sulfhydryl oxidase is the presence of a FAD containing redox center adjacent to the conserved CxxC motif in the ERV1/ALR domain and the dependence on flavin for its enzymatic activity. Mutation of either of the conserved cysteines into serine will inactivate this enzyme [5]. To determine if zebrafish Alr is also a flavin-dependent sulfhydryl oxidase, we expressed and purified recombinant zebrafish Alr and the CxxC mutant Alr^C131S^ proteins from *E. coli* under native condition. The purified recombinant Alr proteins were relatively pure as shown in coomassie blue stained SDS-PAGE gel (Fig. 6A). In the presence of the reducing agent DTT, a single band around 23 kDa is observed in both Alr and Alr^C131S^, consistent to the predicted monomer size. In the absence of DTT, the monomer band disappeared; instead, multiple dimeric bands were detected between the 40–46 kD range. This result indicates that zebrafish Alr also exists as dimer, similar to its human and yeast counterparts [39], [41]. Mutation of cysteine in the C-terminal CxxC motif does not disrupt dimerization.

Zebrafish Alr also binds FAD as determined by spectroscopic absorption, showing two distinct peaks at 360 nm and 450 nm characteristic of FAD (Fig. 6B). The loading of FAD to the monomeric Alr is lower than the expected ratio of 1∶1, possibly due to lower binding efficiency of the protein preparation condition used.

DTT has been shown to be a very good model substrate for flavin-dependent sulfhydryl oxidases, compared to reduced proteins and monothiol molecules [45], [46]. Zebrafish Alr oxidized DTT efficiently, while Alr^C131S^ completely lost this activity (Fig. 6C). Thus, zebrafish Alr is a sulfhydryl oxidase that relies on the proximal CxxC motif for its enzymatic activity. Similar to other ALRs, zebrafish Alr showed almost no detectable activity towards reduced lysozyme and monothiol molecule such as reduced glutathione (data not shown).

### Overexpression of *alr* promotes liver growth and rescues the liver growth defects of *alr* morphants

To determine if the sulfhydryl oxidase activity is important for Alr's function in zebrafish hepatogenesis, we performed overexpression and morphant-rescue studies by microinjecting *in vitro* transcribed *alr* mRNA into 1–2 cell stage embryos. As liver organogenesis is a relatively late developmental event for mRNA overexpression study, we first tested the lifespan of the microinjected *alr* mRNA/protein by injecting the Alr-EGFP fusion mRNA generated from the pCS2+ expression vector. The maturation time of the fusion protein is as fast as EGFP alone [47], with green fluorescence become visible 2–3 h after mRNA injection (data not shown). The green fluorescence is strongest within 30 hpf, after which the signal started to decrease. From 3 dpf onwards, the green fluorescence is no longer visible. Therefore, embryos at 48 hpf were used for liver organogenesis analysis by *prox1* WISH. At this stage, a clear small-liver phenotype can be observed in *alr* morphants and Alr produced from the microinjected mRNA are still present (Fig. 3).

**Figure 7:**
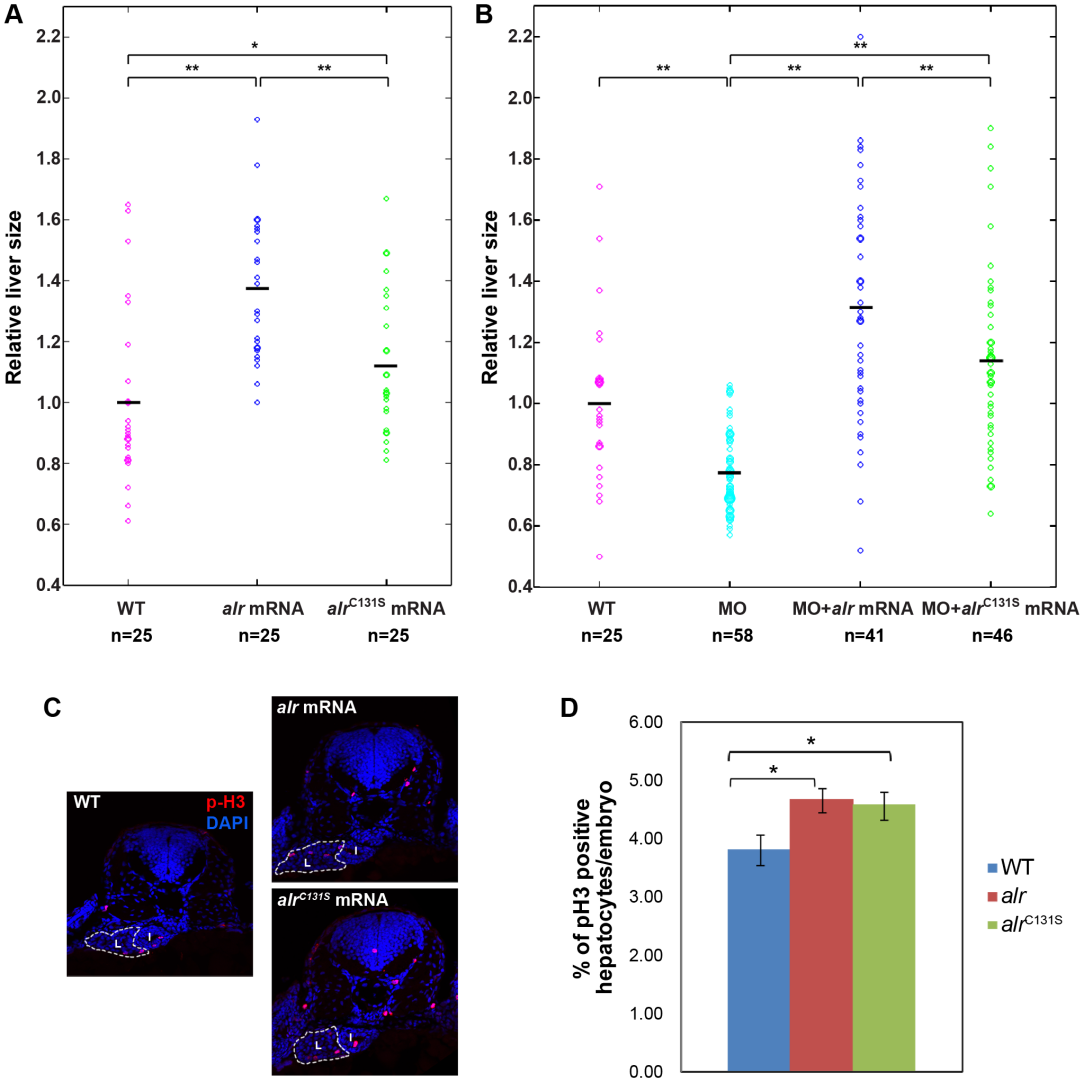
*alr* overexpression promotes liver growth and rescues *alr* morphants. A. Overexpression of both *alr* and enzyme-inactive mutant *alr* ^C131S^ can promote liver growth. But the wild type *alr* is more efficient than *alr* ^C131S^ mutant. The mean liver sizes (means ± SD) are: WT (1.00±0.28), *alr* mRNA (1.37±0.23) and *alr* ^C131S^ mRNA (1.13±0.23). B. Both *alr* and *alr* ^C131S^ overexpression can rescue *alr* morphant and restore liver size. The mean liver sizes (means ± SD) are: WT (1.00±0.25), MO (0.78±0.13), MO+*alr* mRNA (1.30±0.37) and MO+*alr* ^C131S^ mRNA (1.14±0.30). The black line in the middle of scattered dots indicates the mean liver size in that group. The brackets on top indicate the respective two samples compared by student's t-test. n, number of embryo analyzed. *, p<0.05; **, p<0.01. C and D. Overexpression of *alr* and *alr* ^C131S^ promote liver growth by promoting hepatocyte proliferation. Hepatocyte proliferation demonstrated by immunofluorescent staining of proliferation marker p-H3 in 48 hpf embryos (red color). The tissue sections were counterstained with DAPI (blue) to label nucleus. I: intestine; L: liver. Dash line circles the boundary of liver. *, p<0.05. Sample number and other details are described in the relevant methods section.

Embryos injected with *alr* mRNA at ≤1.6 ng per embryo developed normally with no gross morphological abnormalities except a mild 1–2 h precociousness in development (data not shown). Notably, overexpression of Alr (at 1.6 ng mRNA/embryo) significantly enhanced liver growth, with embryos in the overexpression group showing a 40% increase in average liver size comparing to WT embryos at 48 hpf as determined by WISH (Fig. 7A). Interestingly, overexpression of the enzymatically inactive mutant Alr^C131S^ also mildly but significantly promoted liver growth. Comparing to WT Alr, the effect of Alr^C131S^ is noticeably weaker (about 15% increase in average liver size) (Fig. 7A). Nevertheless, the liver growth promoting effect is a consistent phenotype.

The small-liver phenotype resulted from E1I1 morpholino injection (a splicing interference morpholino) was effectively rescued by co-injection of either *alr* or *alr^C131S^* mRNA (Fig. 7B). In *alr* morphants, the relative liver size is in the range of 0.6–0.8. Overexpression of either WT or mutant Alr completely restored the liver size in morphants. Moreover, liver sizes in the *alr* mRNA rescued morphants were about 40% larger than WT embryos, and similar to liver sizes in *alr* overexpressed WT embryos (Fig. 7A and 7B). These results together with morpholino knockdown results establish that Alr is a stimulator of liver growth in zebrafish hepatogenesis.

On the other hand, overexpression of the enzyme-inactive Alr^C131S^ promoted liver growth less efficiently comparing to the WT Alr (Fig. 7A). In addition, although Alr^C131S^ effectively rescued the *alr* morphants and restored the small liver to sizes slightly larger than the WT liver (about 15% larger), the average liver size of Alr^C131S^ rescued embryos is obviously smaller than that of the WT Alr rescued embryos (Fig. 7B). It therefore seems that the sulfhydryl oxidase activity of Alr also contributes to liver outgrowth. Our results suggest that zebrafish *alr* may use both enzyme-dependent as well as enzyme-independent pathways to promote liver growth.

We further showed that the enhanced liver growth is through stimulating hepatocyte proliferation as demonstrated by p-H3 staining (Fig. 7, C and D).

## Discussion

It is hypothesized that genes involved in liver regeneration may also be involved in embryonic liver development. However, to date this has only been documented in a couple of genes. One example, *uhrf1* gene stimulates both adult liver regeneration as well as embryonic liver outgrowth in zebrafish [18], [48]. ALR is an established hepatotrophic growth factor activated during liver regeneration and specifically stimulates hepatocyte proliferation [4]. However, its role in vertebrate embryonic development has not been examined.

In this study, we demonstrated for the first time that *alr* plays a critical role in liver growth during zebrafish hepatogenesis. We showed that *alr* is temporally and spatially expressed in the developing liver at a high level throughout zebrafish liver organogenesis (Fig. 2). Knockdown of *alr* by morpholino antisense oligonucleotide suppressed liver growth, generating a small-liver phenotype without affecting hepatoblast determination from the anterior endoderm (Fig. 3). We further demonstrated that *alr* promotes liver growth by stimulating hepatocyte proliferation rather than inhibiting apoptosis (Fig. 4). This is in accordance with the findings that apoptosis levels are generally low during this stage of liver development (this work and [35]). It is noted that in mammals an anti-apoptotic function of ALR for adult hepatocytes have been reported [49]. Nevertheless, the minimum apoptosis during normal embryonic liver formation in zebrafish possibly renders this function of Alr unimportant in this developmental process. Our work demonstrates that *alr* is a new member to the growing list of genes regulating vertebrate hepatogenesis.

We noted that knockdown of *alr* also resulted in a smaller exocrine pancreas (Fig. 3C and 3E). This is correlated with the expression of *alr* in this organ (Fig. S3). It seems that *alr* could be playing a role in the development of exocrine pancreas. Future investigations are required to elucidate the role of *alr* in exocrine pancreas development.

What is unique about ALR is that this protein is not only a hepatic cytokine but also a sulfhydryl oxidase carrying out fundamental redox reactions in cells. The sulfhydryl oxidase activity of the yeast ALR ortholog, ERV1, is essential for the survival of this single cell organism [41], [50]. Recombinant zebrafish Alr protein expressed from *E.coli* also binds FAD and has sulfhydryl oxidase activity (Fig. 6), presenting similar enzymatic characteristics as mammalian ALRs/yeast ERV1.

Through overexpression and morphant-rescue experiments, we demonstrated that the sulfhydryl oxidase activity may not be essential for Alr's function in promoting liver outgrowth during embryonic development. Overexpression of the enzymatically-inactive mutant Alr^C131S^ also promoted liver growth and rescued the small-liver phenotype in *alr* morphants (Fig. 7). Nevertheless, overexpression of Alr^C131S^ promoted liver growth less efficiently comparing to the wild type Alr (Fig. 7A). Furthermore, although Alr^C131S^ effectively rescued the *alr* morphants, the average liver size of Alr^C131S^ rescued embryos is smaller than that of the wild type Alr rescued embryos (Fig. 7B). This suggests that zebrafish *alr* most likely promotes liver growth through both enzyme-dependent as well as enzyme-independent signaling pathways.

Both enzyme-dependent and -independent signaling pathways of ALR have been illustrated in cultured human hepatoma cells. Extracellular ALR can activate the mitogen-activated protein kinase (MAPK) cascade through its cell surface receptor independent of its sulfhydryl oxidase activity [12], [51]. On the other hand, the ability of intracellular ALR to potentiate the activator protein-1 (AP-1) pathway through JAB1 is dependent on its enzymatic function [12]. Alr may use both the enzyme-dependent (through AP-1 pathway) and enzyme-independent signaling pathways (through MAPK pathway) to promote liver growth during hepatogenesis.

Although we did not detect any secreted zebrafish Alr-V5 or Alr-EGFP fusion protein in the media of cultured cells, it is probable that Alr can be released by hepatocyte under specific environmental conditions such as after liver injury. Incidentally, although low level ALR was detected in medium of primary rat hepatocytes by ELISA [9], no secreted ALR could be detected when a rat ALR cDNA expression plasmid was transfected into cultured COS cells by an *in vivo* functional assay [1]. Hence, different detection methods, different cell types used and different environmental conditions may generate varied results in terms of ALR secretion. Therefore, it is highly probable that zebrafish Alr can be secreted during hepatogenesis.

Alternatively, it can be speculated that exogenously introduced Alr^C131S^ may form functional heterodimers with the residual wild-type Alr protein in *alr* morphants, thus partially rescued the morphant phenotype and promoted liver growth. However, in *alr* morphants with liver growth defects, the amount of *alr* mRNA is almost undetectable up to 5 dpf (Fig. 3 and data not shown), suggesting that the amount of Alr-Alr^C131S^ heterodimers would be very low and thus unlikely to be able to restore the liver defect in morphants to wild type level (Alr^C131S^-Alr^C131S^ homodimer is enzymatically inactive). Future study of Alr^C131S^ in an *alr* complete knockout zebrafish (if possible) would help to exclude this second possibility.

There are two isoforms of ALR protein in mammals, with long form contain about an additional N-terminal non-conserved region. Both isoforms contain no signal peptide at the N-terminal. The composition of the secreted ALR that stimulate hepatocyte proliferation is still not very clear up to now [9]. The zebrafish Alr (191 amino acids) is more similar to the long form mammalian ALR in length. Expression of Alr-V5 and Alr-EGFP fusion proteins in cultured human cells and zebrafish liver cells shows that Alr is localized in both the cytosol and mitochondria, but not in the nucleus or the culture medium (Fig. 5). Notably, the secretion of the mammalian ALR into blood circulation is only sharply up-regulated during liver regeneration after partial hepatectomy with concomitant decrease in hepatic ALR protein [9]. The intracellular localization of zebrafish Alr is also similar to the long form of human ALR205. It is believed that the intracellular ALR is present in many cell types and carries out fundamental cellular functions such as promoting disulfide bond formation in proteins, Fe-S cluster formation and cellular Fe homeostasis [4]. Indeed, when injected with high doses of *alr* morpholino, the morphants exhibited severe defects in multiple organs and embryonic death (data not shown). Under low dose morpholino, it is likely that the residual amount of Alr is sufficient for early embryonic development, but not sufficient to support normal liver growth. Accordingly, *alr* morphants only showed a small-liver phenotype, but not completely lack of liver growth. The clear correlation of knockdown levels with different phenotypes and their severeness supports the current model that Alr performs different functions at different cellular locations and developmental stages.

In addition to liver, *alr* is also expressed at high levels ubiquitously in early embryos (before segmentation) and later in the developing brain and pharyngeal arches (Fig. 2). This is consistent with the *alr* sequence-dependent severe developmental defects and early embryonic death when high doses of morpholino were injected. It therefore seems that a low level sulfhydryl oxidase activity of Alr is essential for fundamental cellular survival. Hence the dose-dependent and partial suppression of *alr* function through morpholino-mediated knockdown presented a clear advantage over the gene knockout approach, allowing the identification of a late developmental role of *alr* in vertebrate liver organogenesis. Incidentally, *alr* mutant in Drosophila is recessive lethal and homozygous *alr* mutation leads to developmental arrest in flies [52].

### Conclusion

In this study, we provide several experimental evidences revealing the role of *alr* in vertebrate liver organogenesis. Using knockdown and overexpression approaches, we demonstrated its positive function in promoting liver growth. We further showed that impaired proliferation but not increased apoptosis was the underlying mechanism for the liver growth defect in *alr* morphants. We show that zebrafish Alr naturally exist in dimer form, is also a flavin-linked sulfhydryl oxidase. By combining biochemistry study with developmental biology study, we show that zebrafish *alr* may use both enzyme-dependent and enzyme-independent signaling pathways to promote liver growth during hepatogenesis.

## Supporting Information

Figure S1 **Comparison of ALR protein sequences.** Sequence alignment of ALR proteins were performed using clustalX program. ALR protein sequences used are: NP_005253 (Homo sapiens) (long), NP_075527 (Mus musculus) (long), EDM03859 (Rattus norvegicus) (long), NP_001082855 (Danio rerio), NP_011543 (Saccharomyces cerevisiae). All the cysteines are highlighted in red. In human, mouse and rat, methionines labeled by blue are the starting amino acids of the short form ALR proteins; in zebrafish, the conserved methionine at same position is also highlighted by blue. Grey brackets mark the Erv1/ALR domain. Green brackets indicate the known intra-molecular disulfide bonds while green arrows indicate the cysteines residues that form the inter-molecular disulfide bonds. The conserved Arginines, which correspond to the position of the R194 mutation in human ALR, are highlighted in purple.
(TIF)

Figure S2 **Zebrafish Alr is the ortholog of mammalian ALR and yeast Erv1p.** A. Phylogenetic tree was constructed using MEGA version 4 (Tamura, Dudley, Nei, and Kumar 2007). The branches were validated by bootstrap analysis from 1000 replications, which were represented by percentage in branch nodes. The scale bar under the tree indicates the p-distance. ALR protein sequences used in this analysis are: NP_005253 (Homo sapiens) (long), P55789 (Homo sapiens) (short), NP_075527 (Mus musculus) (long), P56213 (Mus musculus) (short), EDM03859 (Rattus norvegicus) (long), NP_037354 (Rattus norvegicus) (short), XP_414848 (Gallus gallus), AAH97922 (Xenopus laevis), CAF89716 (Tetraodon nigroviridis), NP_001082855 (Danio rerio), NP_608353 (Drosophila melanogaster), NP_490690 (Caenorhabditis elegans), NP_011543 (Saccharomyces cerevisiae) (Erv1p), NP_015362 (Saccharomyces cerevisiae) (Erv2p). B. Synteny analysis of *alr* (*gfer*) with neighbor genes in zebrafish, chicken, mouse and human genomes. Only one copy of *alr* gene was found in the genomes of the four species. Homologous genes are labeled by the same color. Arrow head shows the direction of that gene. Tamura K, Dudley J, Nei M & Kumar S (2007) MEGA4: Molecular Evolutionary Genetics Analysis (MEGA) software version 4.0. Molecular Biology and Evolution 24:1596–1599.
(TIF)

Figure S3 **Expression of** ***alr*** **in zebrafish exocrine pancreas.** Cross-sections of 4 dpf embryos after WISH with *alr* probe were presented. A, cross section of embryo at the position of liver, dash line circles the liver. B, cross section of embryo at the position of anterior pancreas, the pancreas is circled by dash line. Expression of *alr* is found in exocrine pancreas. C, cross section of embryo at the position of posterior pancreas, dash line circles the exocrine pancreas. L: liver; P: pancreas.
(TIF)

Figure S4 **Liver growth is significantly inhibited in** ***alr*** **morphants.** Cryostat section was obtained from 5 dpf *Tg(lfabp:DsRed; elaA:EGFP)* embryos. Red color is from the DsRed expressed under *lfabp* promoter, indicating the liver. Blue color is the nucleus staining by DAPI. Images in the same column are sections from similar anterior-posterior position of liver.
(TIF)

Figure S5 **Hepatocyte apoptosis is not elevated in** ***alr*** **morphants.** A–F, TUNEL assay performed on 4 dpf embryo liver sections. White dashed lines outline the liver. White arrowheads indicate some of the positively stained cells, which are undergoing apoptosis. Very low levels of apoptosis are found in the developing livers of wild type embryos and *alr* morphants. G, DNase treated sample from 30 hpf embryos, as a positive control. H, brain section from 30 hpf embryos, treated by heat shock (39 degree, 1 hour) to induce apoptosis, as a positive control. L: liver; I: intestine. (Yabu et al., 2001) Yabu, T., Todoriki, S., Yamashita, M., 2001. Stress-induced apoptosis by heat shock, UV and γ-ray irradiation in zebrafish embryos detected by increased caspase activity and whole-mount TUNEL staining. Fisheries Science 67, 333–340.
(TIF)

Figure S6 **Cellular localization of Alr-EGFP in zebrafish embryo and cultured cells.** A. Alr-EGFP is mainly localized in the cytoplasm in zebrafish embryo. The plasmid expressing Alr-EGFP fusion protein under the CMV promoter, was injected into zebrafish 1-cell stage embryos and these embryos were fixed at shield stages (6 hpf) and processed for sectioning. The cryo-sections were stained with mouse anti-GFP primary antibody and Alexa Fluor 568 conjugated anti-mouse IgG secondary antibody. DAPI was used to stain nucleus. Red color shows the predominant presence of Alr-EGFP fusion protein in cytoplasm, but not nucleus. B. Alr-EGFP is localized in both the cytosol and mitochondria. HEK293T cells were transfected with Alr-EGFP expressing plasmid. Cell fractionation followed by Western blot using anti-EGFP antibody revealed that Alr-EGFP was localized in both the cytosol and mitochondria in transfected HEK293T cells. The mitochondrial porin voltage-dependent anion channel (VDAC) was used as the mitochondria marker while α-tubulin was used as the cytosolic marker. C. Alr was not secreted outside of cell. Alr-EGFP is detected by anti-GFP antibody Western blot. The β-actin was used as loading control. L, cell lysate; M, conditioned medium.
(TIF)

## References

[1] Hagiya M, Francavilla A, Polimeno L, Ihara I, Sakai H (1994). Cloning and sequence analysis of the rat augmenter of liver regeneration (ALR) gene: expression of biologically active recombinant ALR and demonstration of tissue distribution.. Proc Natl Acad Sci U S A.

[2] Lisowsky T, Weinstat-Saslow DL, Barton N, Reeders ST, Schneider MC (1995). A new human gene located in the PKD1 region of chromosome 16 is a functional homologue to ERV1 of yeast.. Genomics.

[3] Yang X, Xie L, Qiu Z, Wu Z, He F (1997). Human augmenter of liver regeneration: Molecular cloning, biological activity and roles in liver regeneration.. Sci China C Life Sci.

[4] Gatzidou E, Kouraklis G, Theocharis S (2006). Insights on augmenter of liver regeneration cloning and function.. World J Gastroenterol.

[5] Lisowsky T, Lee JE, Polimeno L, Francavilla A, Hofhaus G (2001). Mammalian augmenter of liver regeneration protein is a sulfhydryl oxidase.. Dig Liver Dis.

[6] Mesecke N, Terziyska N, Kozany C, Baumann F, Neupert W (2005). A disulfide relay system in the intermembrane space of mitochondria that mediates protein import.. Cell.

[7] Allen S, Balabanidou V, Sideris DP, Lisowsky T, Tokatlidis K (2005). Erv1 mediates the Mia40-dependent protein import pathway and provides a functional link to the respiratory chain by shuttling electrons to cytochrome c.. J Mol Biol.

[8] Farrell SR, Thorpe C (2005). Augmenter of liver regeneration: a flavin-dependent sulfhydryl oxidase with cytochrome c reductase activity.. Biochemistry.

[9] Gandhi CR, Kuddus R, Subbotin VM, Prelich J, Murase N (1999). A fresh look at augmenter of liver regeneration in rats.. Hepatology.

[10] Wang G, Yang X, Zhang Y, Wang Q, Chen H (1999). Identification and characterization of receptor for mammalian hepatopoietin that is homologous to yeast ERV1.. J Biol Chem.

[11] Lu C, Li Y, Zhao Y, Xing G, Tang F (2002). Intracrine hepatopoietin potentiates AP-1 activity through JAB1 independent of MAPK pathway.. Faseb J.

[12] Chen X, Li Y, Wei K, Li L, Liu W (2003). The potentiation role of hepatopoietin on activator protein-1 is dependent on its sulfhydryl oxidase activity.. J Biol Chem.

[13] Di Fonzo A, Ronchi D, Lodi T, Fassone E, Tigano M (2009). The mitochondrial disulfide relay system protein GFER is mutated in autosomal-recessive myopathy with cataract and combined respiratory-chain deficiency.. Am J Hum Genet.

[14] Daithankar VN, Schaefer SA, Dong M, Bahnson BJ, Thorpe C (2010). Structure of the human sulfhydryl oxidase augmenter of liver regeneration and characterization of a human mutation causing an autosomal recessive myopathy.. Biochemistry.

[15] LaBrecque DR, Pesch LA (1975). Preparation and partial characterization of hepatic regenerative stimulator substance (SS) from rat liver.. J Physiol.

[16] He F, Wu C, Tu Q, Xing G (1993). Human hepatic stimulator substance: a product of gene expression of human fetal liver tissue.. Hepatology.

[17] Chu J, Sadler KC (2009). New school in liver development: lessons from zebrafish.. Hepatology.

[18] Sadler KC, Krahn KN, Gaur NA, Ukomadu C (2007). Liver growth in the embryo and during liver regeneration in zebrafish requires the cell cycle regulator, uhrf1.. Proc Natl Acad Sci U S A.

[19] Field HA, Ober EA, Roeser T, Stainier DY (2003). Formation of the digestive system in zebrafish. I. Liver morphogenesis.. Dev Biol.

[20] Shin D, Shin CH, Tucker J, Ober EA, Rentzsch F (2007). Bmp and Fgf signaling are essential for liver specification in zebrafish.. Development.

[21] Ober EA, Verkade H, Field HA, Stainier DY (2006). Mesodermal Wnt2b signalling positively regulates liver specification.. Nature.

[22] Kimmel CB, Ballard WW, Kimmel SR, Ullmann B, Schilling TF (1995). Stages of embryonic development of the zebrafish.. Dev Dyn.

[23] Westerfield M (1995). The Zebrafish Book.

[24] Farooq M, Sulochana KN, Pan X, To J, Sheng D (2008). Histone deacetylase 3 (hdac3) is specifically required for liver development in zebrafish.. Dev Biol.

[25] Ghosh C, Zhou YL, Collodi P (1994). Derivation and characterization of a zebrafish liver cell line.. Cell Biology and Toxicology.

[26] Thasler WE, Schlott T, Thelen P, Hellerbrand C, Bataille F (2005). Expression of augmenter of liver regeneration (ALR) in human liver cirrhosis and carcinoma.. Histopathology.

[27] Tanigawa K, Sakaida I, Masuhara M, Hagiya M, Okita K (2000). Augmenter of liver regeneration (ALR) may promote liver regeneration by reducing natural killer (NK) cell activity in human liver diseases.. J Gastroenterol.

[28] Cao Y, Fu YL, Yu M, Yue PB, Ge CH (2009). Human augmenter of liver regeneration is important for hepatoma cell viability and resistance to radiation-induced oxidative stress.. Free Radic Biol Med.

[29] Thirunavukkarasu C, Wang LF, Harvey SA, Watkins SC, Chaillet JR (2008). Augmenter of liver regeneration: an important intracellular survival factor for hepatocytes.. J Hepatol.

[30] Liatsos GD, Mykoniatis MG, Margeli A, Liakos AA, Theocharis SE (2003). Effect of acute ethanol exposure on hepatic stimulator substance (HSS) levels during liver regeneration: protective function of HSS.. Dig Dis Sci.

[31] Kondili VG, Tzirogiannis KN, Androutsos CD, Papadimas GK, Demonakou MD (2005). The hepatoprotective effect of hepatic stimulator substance (HSS) against liver regeneration arrest induced by acute ethanol intoxication.. Dig Dis Sci.

[32] Gandhi CR, Murase N, Starzl TE (2009). Cholera toxin-sensitive GTP-binding protein-coupled activation of augmenter of liver regeneration (ALR) receptor and its function in rat kupffer cells.. J Cell Physiol.

[33] Passeri MJ, Cinaroglu A, Gao C, Sadler KC (2009). Hepatic steatosis in response to acute alcohol exposure in zebrafish requires sterol regulatory element binding protein activation.. Hepatology.

[34] Korzh S, Emelyanov A, Korzh V (2001). Developmental analysis of ceruloplasmin gene and liver formation in zebrafish.. Mech Dev.

[35] Chen J, Ruan H, Ng SM, Gao C, Soo HM (2005). Loss of function of def selectively up-regulates Delta113p53 expression to arrest expansion growth of digestive organs in zebrafish.. Genes Dev.

[36] Yabu T, Todoriki S, Yamashita M (2001). Stress-induced apoptosis by heat shock, UV and γ-ray irradiation in zebrafish embryos detected by increased caspase activity and whole-mount TUNEL staining.. Fisheries Science.

[37] Hofhaus G, Stein G, Polimeno L, Francavilla A, Lisowsky T (1999). Highly divergent amino termini of the homologous human ALR and yeast scERV1 gene products define species specific differences in cellular localization.. Eur J Cell Biol.

[38] Lange H, Lisowsky T, Gerber J, Muhlenhoff U, Kispal G (2001). An essential function of the mitochondrial sulfhydryl oxidase Erv1p/ALR in the maturation of cytosolic Fe/S proteins.. EMBO Rep.

[39] Li Y, Wei K, Lu C, Li M, Xing G (2002). Identification of hepatopoietin dimerization, its interacting regions and alternative splicing of its transcription.. Eur J Biochem.

[40] Lu J, Xu WX, Zhan YQ, Cui XL, Cai WM (2002). Identification and characterization of a novel isoform of hepatopoietin.. World J Gastroenterol.

[41] Lee J, Hofhaus G, Lisowsky T (2000). Erv1p from Saccharomyces cerevisiae is a FAD-linked sulfhydryl oxidase.. FEBS Lett.

[42] Levitan A, Danon A, Lisowsky T (2004). Unique features of plant mitochondrial sulfhydryl oxidase.. J Biol Chem.

[43] Tokatlidis K (2005). A disulfide relay system in mitochondria.. Cell.

[44] Bihlmaier K, Mesecke N, Terziyska N, Bien M, Hell K (2007). The disulfide relay system of mitochondria is connected to the respiratory chain.. J Cell Biol.

[45] Wang W, Winther JR, Thorpe C (2007). Erv2p: characterization of the redox behavior of a yeast sulfhydryl oxidase.. Biochemistry.

[46] Daithankar VN, Farrell SR, Thorpe C (2009). Augmenter of liver regeneration: substrate specificity of a flavin-dependent oxidoreductase from the mitochondrial intermembrane space.. Biochemistry.

[47] Wiedenmann J, Oswald F, Nienhaus GU (2009). Fluorescent proteins for live cell imaging: opportunities, limitations, and challenges.. IUBMB Life.

[48] Wang CP, Zhou L, Su SH, Chen Y, Lu YY (2006). Augmenter of liver regeneration promotes hepatocyte proliferation induced by Kupffer cells.. World J Gastroenterol.

[49] Ilowski M, Kleespies A, de Toni EN, Donabauer B, Jauch KW (2011). Augmenter of liver regeneration (ALR) protects human hepatocytes against apoptosis.. Biochem Biophys Res Commun.

[50] Lisowsky T (1992). Dual function of a new nuclear gene for oxidative phosphorylation and vegetative growth in yeast.. Mol Gen Genet.

[51] Li Y, Li M, Xing G, Hu Z, Wang Q (2000). Stimulation of the mitogen-activated protein kinase cascade and tyrosine phosphorylation of the epidermal growth factor receptor by hepatopoietin.. J Biol Chem.

[52] McClure KD, Sustar A, Schubiger G (2008). Three genes control the timing, the site and the size of blastema formation in Drosophila.. Dev Biol.

