# Supplementary figures and images for: Augmenter of Liver Regeneration (alr) Promotes Liver Outgrowth during Zebrafish Hepatogenesis
Source: PLoS One. 2012 Jan 26;7(1):e30835. doi: 10.1371/journal.pone.0030835 (PMC3266923; doi:10.1371/journal.pone.0030835)

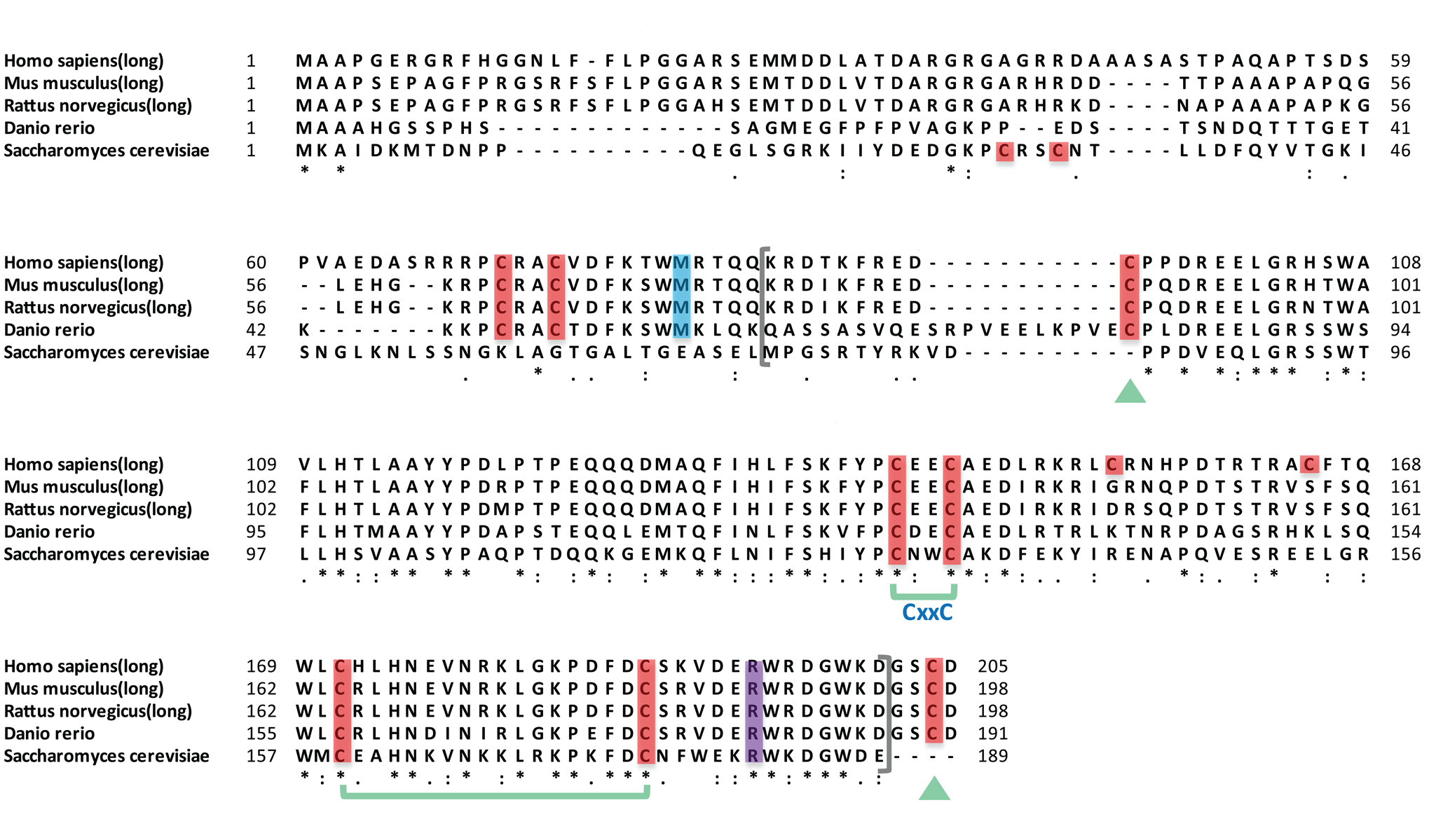

Supplement: Figure S1 — Comparison of ALR protein sequences. Sequence alignment of ALR proteins were performed using clustalX program. ALR protein sequences used are: NP_005253 (Homo sapiens) (long), NP_075527 (Mus musculus) (long), EDM03859 (Rattus norvegicus) (long), NP_001082855 (Danio rerio), NP_011543 (Saccharomyces cerevisiae). All the cysteines are highlighted in red. In human, mouse and rat, methionines labeled by blue are the starting amino acids of the short form ALR proteins; in zebrafish, the conserved methionine at same position is also highlighted by blue. Grey brackets mark the Erv1/ALR domain. Green brackets indicate the known intra-molecular disulfide bonds while green arrows indicate the cysteines residues that form the inter-molecular disulfide bonds. The conserved Arginines, which correspond to the position of the R194 mutation in human ALR, are highlighted in purple. (TIF) [file pone.0030835.s001.tif]

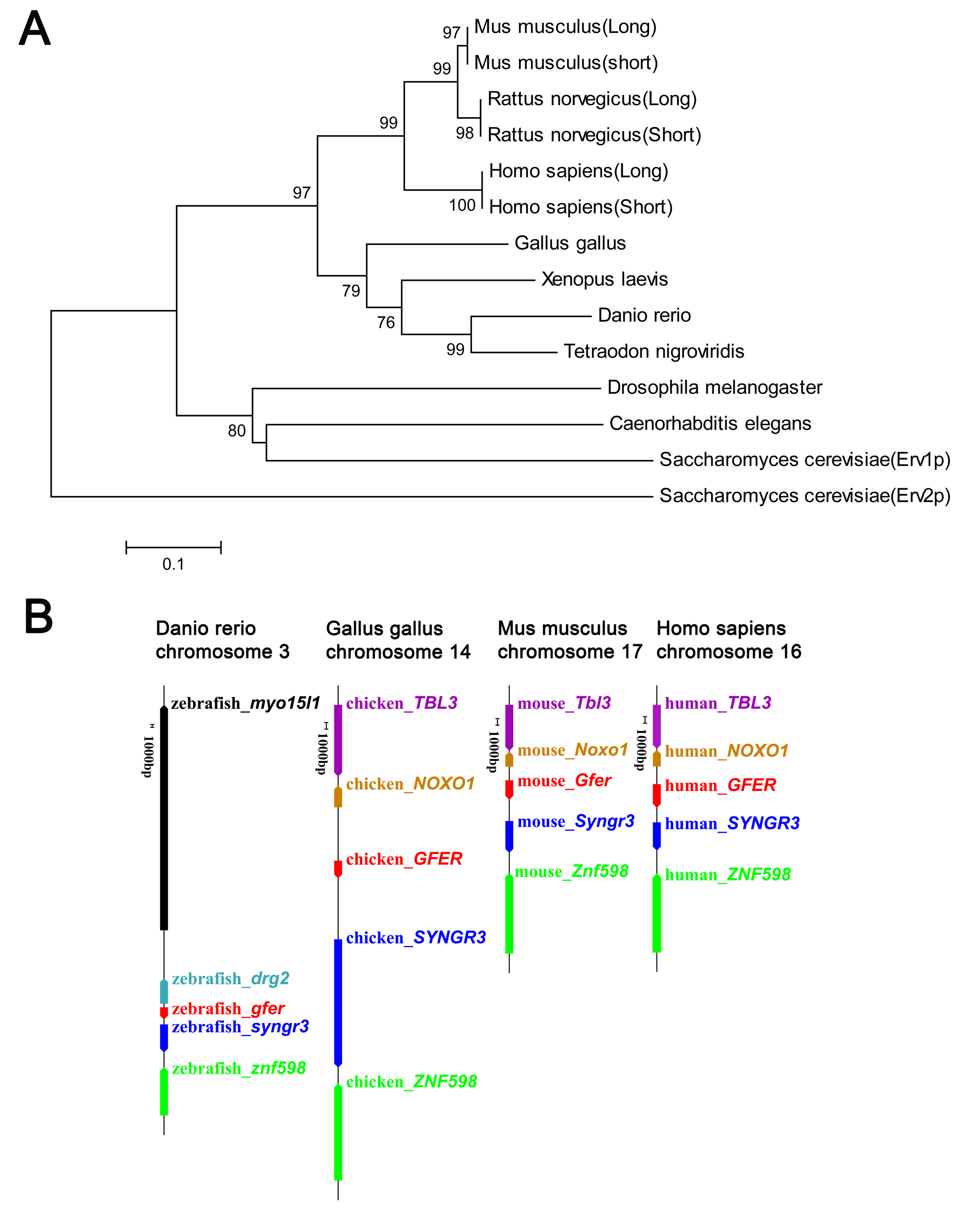

Supplement: Figure S2 — Zebrafish Alr is the ortholog of mammalian ALR and yeast Erv1p. A. Phylogenetic tree was constructed using MEGA version 4 (Tamura, Dudley, Nei, and Kumar 2007). The branches were validated by bootstrap analysis from 1000 replications, which were represented by percentage in branch nodes. The scale bar under the tree indicates the p-distance. ALR protein sequences used in this analysis are: NP_005253 (Homo sapiens) (long), P55789 (Homo sapiens) (short), NP_075527 (Mus musculus) (long), P56213 (Mus musculus) (short), EDM03859 (Rattus norvegicus) (long), NP_037354 (Rattus norvegicus) (short), XP_414848 (Gallus gallus), AAH97922 (Xenopus laevis), CAF89716 (Tetraodon nigroviridis), NP_001082855 (Danio rerio), NP_608353 (Drosophila melanogaster), NP_490690 (Caenorhabditis elegans), NP_011543 (Saccharomyces cerevisiae) (Erv1p), NP_015362 (Saccharomyces cerevisiae) (Erv2p). B. Synteny analysis of alr (gfer) with neighbor genes in zebrafish, chicken, mouse and human genomes. Only one copy of alr gene was found in the genomes of the four species. Homologous genes are labeled by the same color. Arrow head shows the direction of that gene. Tamura K, Dudley J, Nei M & Kumar S (2007) MEGA4: Molecular Evolutionary Genetics Analysis (MEGA) software version 4.0. Molecular Biology and Evolution 24:1596–1599. (TIF) [file pone.0030835.s002.tif]

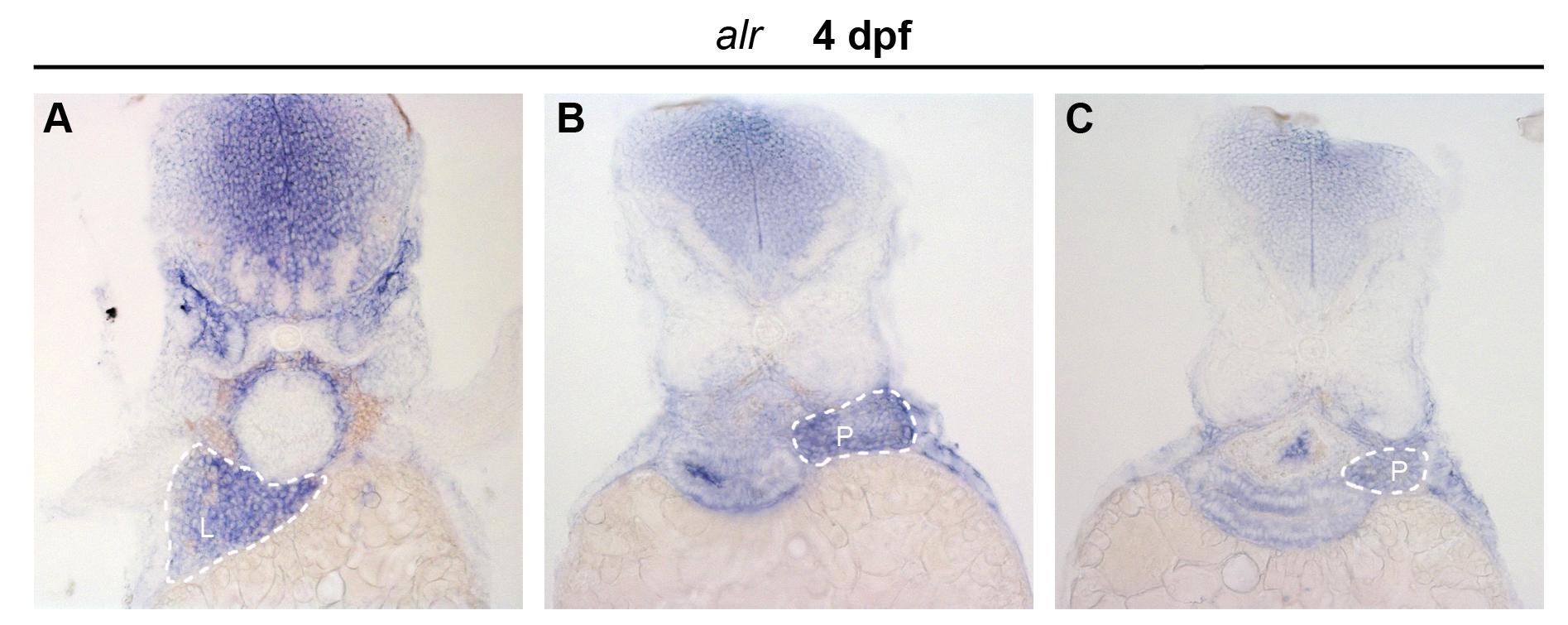

Supplement: Figure S3 — Expression of alr in zebrafish exocrine pancreas. Cross-sections of 4 dpf embryos after WISH with alr probe were presented. A, cross section of embryo at the position of liver, dash line circles the liver. B, cross section of embryo at the position of anterior pancreas, the pancreas is circled by dash line. Expression of alr is found in exocrine pancreas. C, cross section of embryo at the position of posterior pancreas, dash line circles the exocrine pancreas. L: liver; P: pancreas. (TIF) [file pone.0030835.s003.tif]

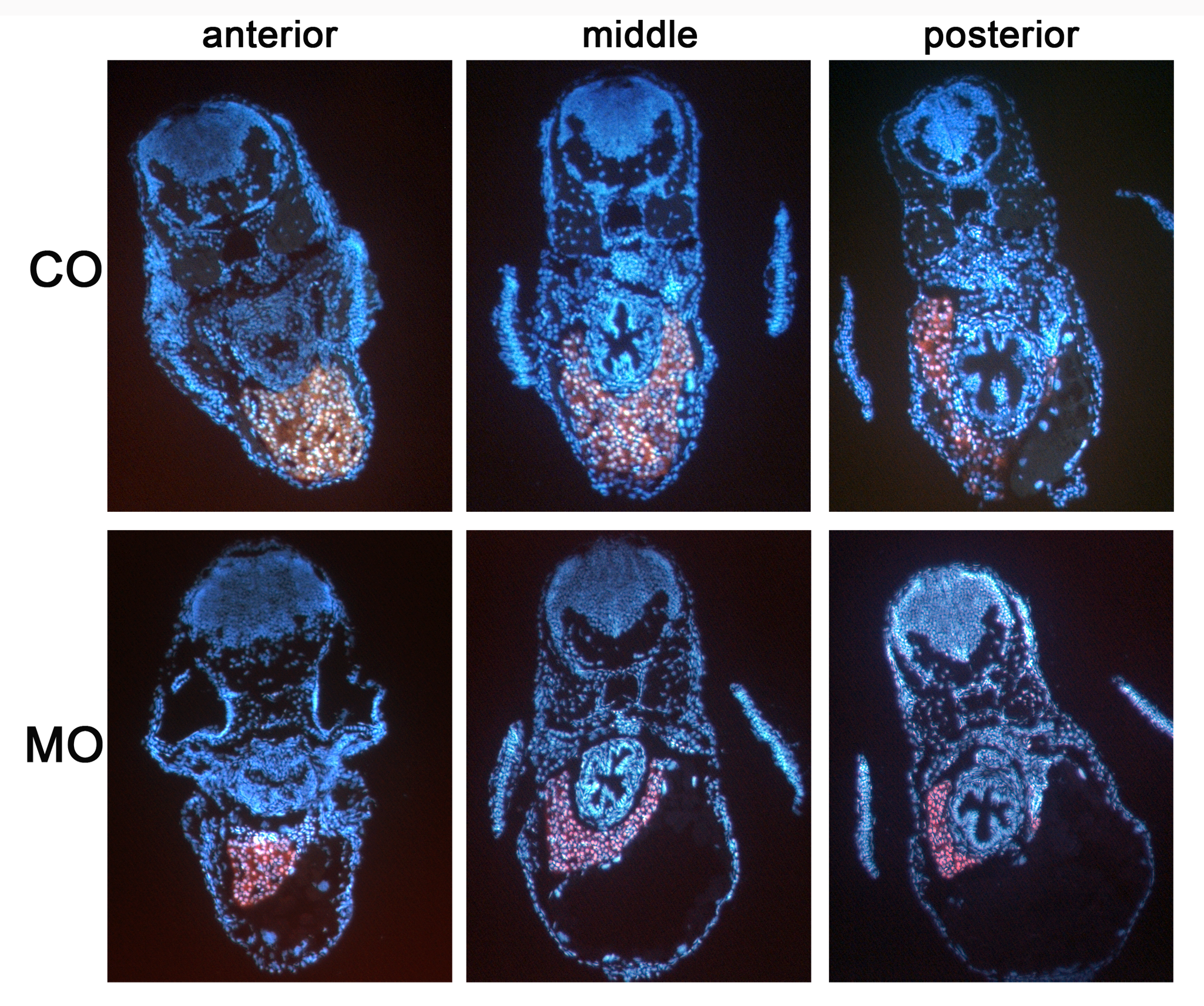

Supplement: Figure S4 — Liver growth is significantly inhibited in alr morphants. Cryostat section was obtained from 5 dpf Tg(lfabp:DsRed; elaA:EGFP) embryos. Red color is from the DsRed expressed under lfabp promoter, indicating the liver. Blue color is the nucleus staining by DAPI. Images in the same column are sections from similar anterior-posterior position of liver. (TIF) [file pone.0030835.s004.tif]

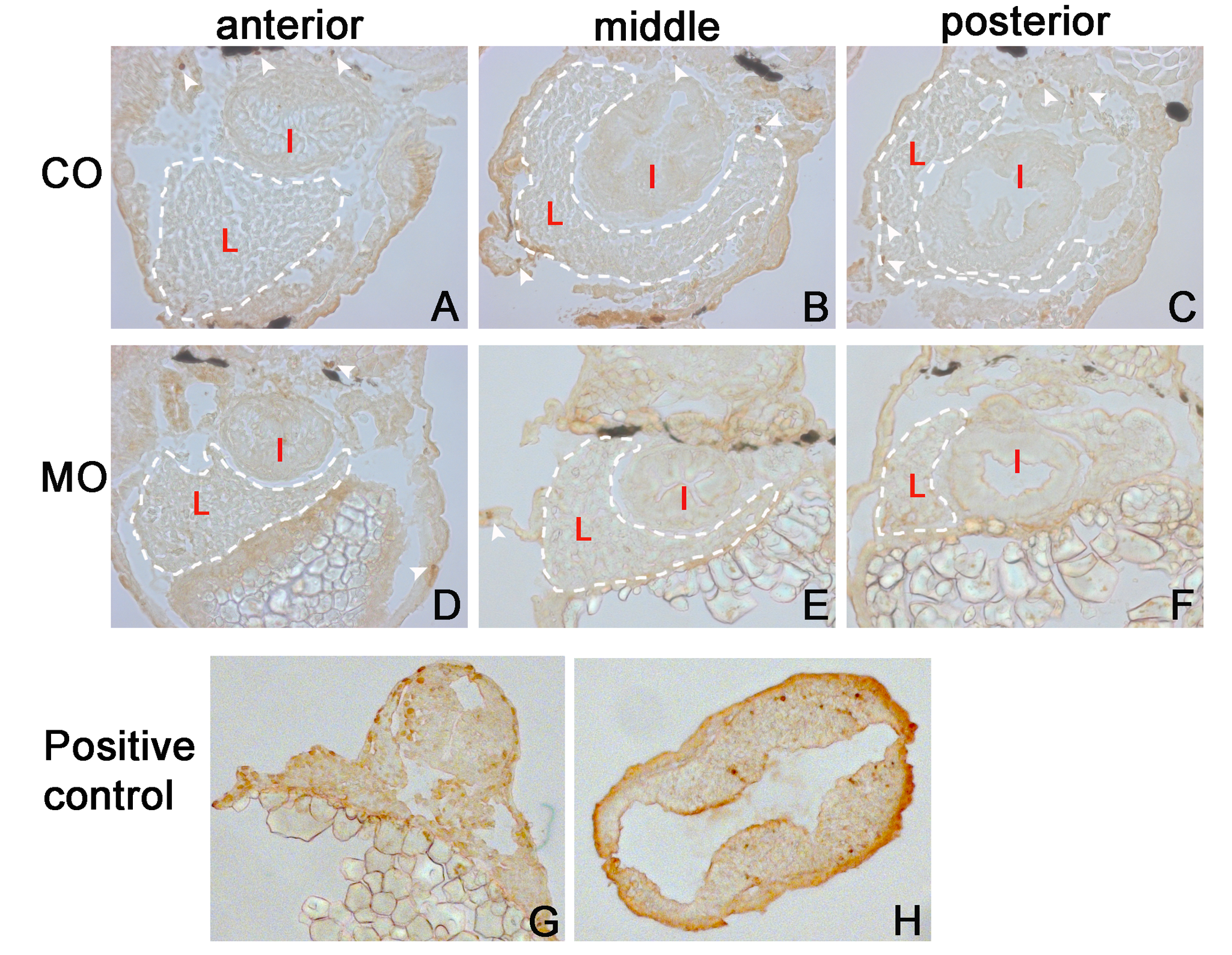

Supplement: Figure S5 — Hepatocyte apoptosis is not elevated in alr morphants. A–F, TUNEL assay performed on 4 dpf embryo liver sections. White dashed lines outline the liver. White arrowheads indicate some of the positively stained cells, which are undergoing apoptosis. Very low levels of apoptosis are found in the developing livers of wild type embryos and alr morphants. G, DNase treated sample from 30 hpf embryos, as a positive control. H, brain section from 30 hpf embryos, treated by heat shock (39 degree, 1 hour) to induce apoptosis, as a positive control. L: liver; I: intestine. (Yabu et al., 2001) Yabu, T., Todoriki, S., Yamashita, M., 2001. Stress-induced apoptosis by heat shock, UV and γ-ray irradiation in zebrafish embryos detected by increased caspase activity and whole-mount TUNEL staining. Fisheries Science 67, 333–340. (TIF) [file pone.0030835.s005.tif]

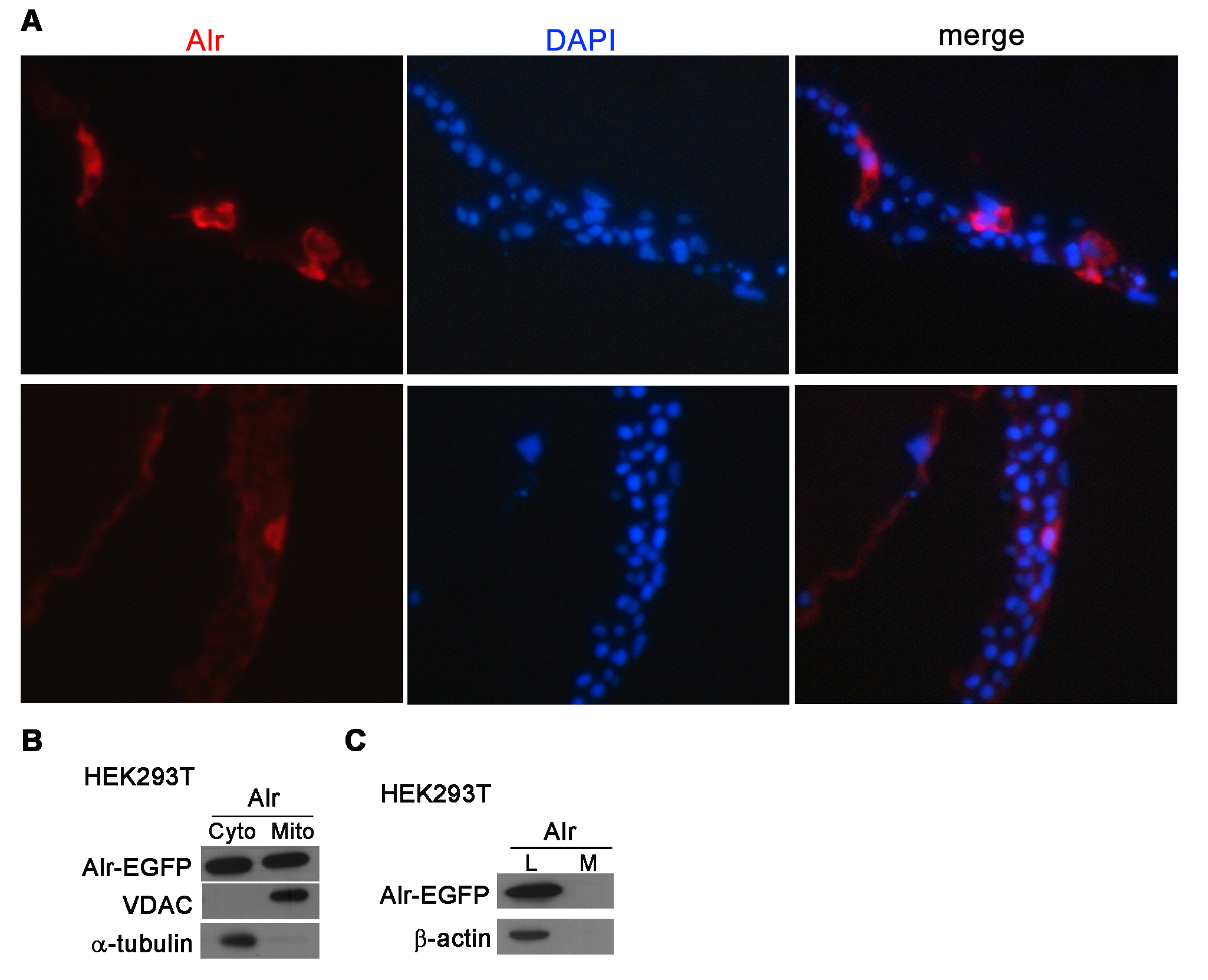

Supplement: Figure S6 — Cellular localization of Alr-EGFP in zebrafish embryo and cultured cells. A. Alr-EGFP is mainly localized in the cytoplasm in zebrafish embryo. The plasmid expressing Alr-EGFP fusion protein under the CMV promoter, was injected into zebrafish 1-cell stage embryos and these embryos were fixed at shield stages (6 hpf) and processed for sectioning. The cryo-sections were stained with mouse anti-GFP primary antibody and Alexa Fluor 568 conjugated anti-mouse IgG secondary antibody. DAPI was used to stain nucleus. Red color shows the predominant presence of Alr-EGFP fusion protein in cytoplasm, but not nucleus. B. Alr-EGFP is localized in both the cytosol and mitochondria. HEK293T cells were transfected with Alr-EGFP expressing plasmid. Cell fractionation followed by Western blot using anti-EGFP antibody revealed that Alr-EGFP was localized in both the cytosol and mitochondria in transfected HEK293T cells. The mitochondrial porin voltage-dependent anion channel (VDAC) was used as the mitochondria marker while α-tubulin was used as the cytosolic marker. C. Alr was not secreted outside of cell. Alr-EGFP is detected by anti-GFP antibody Western blot. The β-actin was used as loading control. L, cell lysate; M, conditioned medium. (TIF) [file pone.0030835.s006.tif]
